# Systematic analysis of siRNA and mRNA features impacting fully chemically modified siRNA efficacy

**DOI:** 10.1093/nar/gkaf479

**Published:** 2025-06-23

**Authors:** Sarah M Davis, Samuel Hildebrand, Hannah J MacMillan, Kathryn R Monopoli, Julianna Buchwald, Jacquelyn Sousa, David Cooper, Socheata Ly, Dimas Echeverria, Nicholas McHugh, Chantal Ferguson, Andrew Coles, Vignesh N Hariharan, Daniel O’Reilly, Qi Tang, Raymond Furgal, Ken Yamada, Julia F Alterman, James W Gilbert, Emily Knox, Yamilett Pineda, Caitlyn N Weston, Christina E Baer, Athma A Pai, Anastasia Khvorova

**Affiliations:** Morningside Graduate School of Biomedical Sciences, T.H. Chan School of Medicine, Interdisciplinary Graduate Program, RNA Therapeutics Institute, Program in Molecular Medicine, Systems Biology, Biochemistry and Molecular Biotechnology, Microbiology and Physiological Systems, MD/PhD Program, University of Massachusetts Chan Medical School, Worcester, MA 01655, United States; Morningside Graduate School of Biomedical Sciences, T.H. Chan School of Medicine, Interdisciplinary Graduate Program, RNA Therapeutics Institute, Program in Molecular Medicine, Systems Biology, Biochemistry and Molecular Biotechnology, Microbiology and Physiological Systems, MD/PhD Program, University of Massachusetts Chan Medical School, Worcester, MA 01655, United States; Morningside Graduate School of Biomedical Sciences, T.H. Chan School of Medicine, Interdisciplinary Graduate Program, RNA Therapeutics Institute, Program in Molecular Medicine, Systems Biology, Biochemistry and Molecular Biotechnology, Microbiology and Physiological Systems, MD/PhD Program, University of Massachusetts Chan Medical School, Worcester, MA 01655, United States; Morningside Graduate School of Biomedical Sciences, T.H. Chan School of Medicine, Interdisciplinary Graduate Program, RNA Therapeutics Institute, Program in Molecular Medicine, Systems Biology, Biochemistry and Molecular Biotechnology, Microbiology and Physiological Systems, MD/PhD Program, University of Massachusetts Chan Medical School, Worcester, MA 01655, United States; Quantitative and Computational Biosciences and Bioengineering Program, University of Massachusetts Chan Medical School and Worcester Polytechnic Institute, Worcester, MA 01655, United States; Morningside Graduate School of Biomedical Sciences, T.H. Chan School of Medicine, Interdisciplinary Graduate Program, RNA Therapeutics Institute, Program in Molecular Medicine, Systems Biology, Biochemistry and Molecular Biotechnology, Microbiology and Physiological Systems, MD/PhD Program, University of Massachusetts Chan Medical School, Worcester, MA 01655, United States; Morningside Graduate School of Biomedical Sciences, T.H. Chan School of Medicine, Interdisciplinary Graduate Program, RNA Therapeutics Institute, Program in Molecular Medicine, Systems Biology, Biochemistry and Molecular Biotechnology, Microbiology and Physiological Systems, MD/PhD Program, University of Massachusetts Chan Medical School, Worcester, MA 01655, United States; Morningside Graduate School of Biomedical Sciences, T.H. Chan School of Medicine, Interdisciplinary Graduate Program, RNA Therapeutics Institute, Program in Molecular Medicine, Systems Biology, Biochemistry and Molecular Biotechnology, Microbiology and Physiological Systems, MD/PhD Program, University of Massachusetts Chan Medical School, Worcester, MA 01655, United States; Morningside Graduate School of Biomedical Sciences, T.H. Chan School of Medicine, Interdisciplinary Graduate Program, RNA Therapeutics Institute, Program in Molecular Medicine, Systems Biology, Biochemistry and Molecular Biotechnology, Microbiology and Physiological Systems, MD/PhD Program, University of Massachusetts Chan Medical School, Worcester, MA 01655, United States; Morningside Graduate School of Biomedical Sciences, T.H. Chan School of Medicine, Interdisciplinary Graduate Program, RNA Therapeutics Institute, Program in Molecular Medicine, Systems Biology, Biochemistry and Molecular Biotechnology, Microbiology and Physiological Systems, MD/PhD Program, University of Massachusetts Chan Medical School, Worcester, MA 01655, United States; Morningside Graduate School of Biomedical Sciences, T.H. Chan School of Medicine, Interdisciplinary Graduate Program, RNA Therapeutics Institute, Program in Molecular Medicine, Systems Biology, Biochemistry and Molecular Biotechnology, Microbiology and Physiological Systems, MD/PhD Program, University of Massachusetts Chan Medical School, Worcester, MA 01655, United States; Morningside Graduate School of Biomedical Sciences, T.H. Chan School of Medicine, Interdisciplinary Graduate Program, RNA Therapeutics Institute, Program in Molecular Medicine, Systems Biology, Biochemistry and Molecular Biotechnology, Microbiology and Physiological Systems, MD/PhD Program, University of Massachusetts Chan Medical School, Worcester, MA 01655, United States; Morningside Graduate School of Biomedical Sciences, T.H. Chan School of Medicine, Interdisciplinary Graduate Program, RNA Therapeutics Institute, Program in Molecular Medicine, Systems Biology, Biochemistry and Molecular Biotechnology, Microbiology and Physiological Systems, MD/PhD Program, University of Massachusetts Chan Medical School, Worcester, MA 01655, United States; Morningside Graduate School of Biomedical Sciences, T.H. Chan School of Medicine, Interdisciplinary Graduate Program, RNA Therapeutics Institute, Program in Molecular Medicine, Systems Biology, Biochemistry and Molecular Biotechnology, Microbiology and Physiological Systems, MD/PhD Program, University of Massachusetts Chan Medical School, Worcester, MA 01655, United States; Morningside Graduate School of Biomedical Sciences, T.H. Chan School of Medicine, Interdisciplinary Graduate Program, RNA Therapeutics Institute, Program in Molecular Medicine, Systems Biology, Biochemistry and Molecular Biotechnology, Microbiology and Physiological Systems, MD/PhD Program, University of Massachusetts Chan Medical School, Worcester, MA 01655, United States; Morningside Graduate School of Biomedical Sciences, T.H. Chan School of Medicine, Interdisciplinary Graduate Program, RNA Therapeutics Institute, Program in Molecular Medicine, Systems Biology, Biochemistry and Molecular Biotechnology, Microbiology and Physiological Systems, MD/PhD Program, University of Massachusetts Chan Medical School, Worcester, MA 01655, United States; Morningside Graduate School of Biomedical Sciences, T.H. Chan School of Medicine, Interdisciplinary Graduate Program, RNA Therapeutics Institute, Program in Molecular Medicine, Systems Biology, Biochemistry and Molecular Biotechnology, Microbiology and Physiological Systems, MD/PhD Program, University of Massachusetts Chan Medical School, Worcester, MA 01655, United States; Morningside Graduate School of Biomedical Sciences, T.H. Chan School of Medicine, Interdisciplinary Graduate Program, RNA Therapeutics Institute, Program in Molecular Medicine, Systems Biology, Biochemistry and Molecular Biotechnology, Microbiology and Physiological Systems, MD/PhD Program, University of Massachusetts Chan Medical School, Worcester, MA 01655, United States; Morningside Graduate School of Biomedical Sciences, T.H. Chan School of Medicine, Interdisciplinary Graduate Program, RNA Therapeutics Institute, Program in Molecular Medicine, Systems Biology, Biochemistry and Molecular Biotechnology, Microbiology and Physiological Systems, MD/PhD Program, University of Massachusetts Chan Medical School, Worcester, MA 01655, United States; Morningside Graduate School of Biomedical Sciences, T.H. Chan School of Medicine, Interdisciplinary Graduate Program, RNA Therapeutics Institute, Program in Molecular Medicine, Systems Biology, Biochemistry and Molecular Biotechnology, Microbiology and Physiological Systems, MD/PhD Program, University of Massachusetts Chan Medical School, Worcester, MA 01655, United States; Morningside Graduate School of Biomedical Sciences, T.H. Chan School of Medicine, Interdisciplinary Graduate Program, RNA Therapeutics Institute, Program in Molecular Medicine, Systems Biology, Biochemistry and Molecular Biotechnology, Microbiology and Physiological Systems, MD/PhD Program, University of Massachusetts Chan Medical School, Worcester, MA 01655, United States; Morningside Graduate School of Biomedical Sciences, T.H. Chan School of Medicine, Interdisciplinary Graduate Program, RNA Therapeutics Institute, Program in Molecular Medicine, Systems Biology, Biochemistry and Molecular Biotechnology, Microbiology and Physiological Systems, MD/PhD Program, University of Massachusetts Chan Medical School, Worcester, MA 01655, United States; Morningside Graduate School of Biomedical Sciences, T.H. Chan School of Medicine, Interdisciplinary Graduate Program, RNA Therapeutics Institute, Program in Molecular Medicine, Systems Biology, Biochemistry and Molecular Biotechnology, Microbiology and Physiological Systems, MD/PhD Program, University of Massachusetts Chan Medical School, Worcester, MA 01655, United States; Morningside Graduate School of Biomedical Sciences, T.H. Chan School of Medicine, Interdisciplinary Graduate Program, RNA Therapeutics Institute, Program in Molecular Medicine, Systems Biology, Biochemistry and Molecular Biotechnology, Microbiology and Physiological Systems, MD/PhD Program, University of Massachusetts Chan Medical School, Worcester, MA 01655, United States; Morningside Graduate School of Biomedical Sciences, T.H. Chan School of Medicine, Interdisciplinary Graduate Program, RNA Therapeutics Institute, Program in Molecular Medicine, Systems Biology, Biochemistry and Molecular Biotechnology, Microbiology and Physiological Systems, MD/PhD Program, University of Massachusetts Chan Medical School, Worcester, MA 01655, United States; Morningside Graduate School of Biomedical Sciences, T.H. Chan School of Medicine, Interdisciplinary Graduate Program, RNA Therapeutics Institute, Program in Molecular Medicine, Systems Biology, Biochemistry and Molecular Biotechnology, Microbiology and Physiological Systems, MD/PhD Program, University of Massachusetts Chan Medical School, Worcester, MA 01655, United States

## Abstract

Chemically modified small interfering RNAs (siRNAs) are a promising drug class that silences disease-causing genes via mRNA degradation. Both siRNA-specific features (e.g. sequence, modification pattern, and structure) and target mRNA-specific factors contribute to observed efficacy. Systematically defining the relative contributions of siRNA sequence, structure, and modification pattern versus the native context of the target mRNA is necessary to inform design considerations and facilitate the widespread application of this therapeutic platform. To address this, we synthesized a panel of ∼1260 differentially modified siRNAs and evaluated their silencing efficiency against therapeutically relevant mRNAs (*APP*, *BACE1*, *MAPT*, and *SNCA*) using both reporter-based and native expression assays. Our results demonstrate that the siRNA modification pattern (e.g. level of 2′-*O*-methyl content) significantly impacts efficacy, while structural features (e.g. symmetric versus asymmetric configurations) do not. Furthermore, we observed substantial differences in the number of effective siRNAs identified per target. These target-specific differences in hit rates are largely mitigated when efficacy is tested in the context of a reporter assay, confirming that native mRNA-specific features influence siRNA performance. Key target-specific factors, including exon usage, polyadenylation site selection, and ribosomal occupancy, partially explained efficacy variability. These insights led to a proposed framework of parameters for optimizing therapeutic siRNA design.

## Graphical Abstract

**Graphical Abstract:**
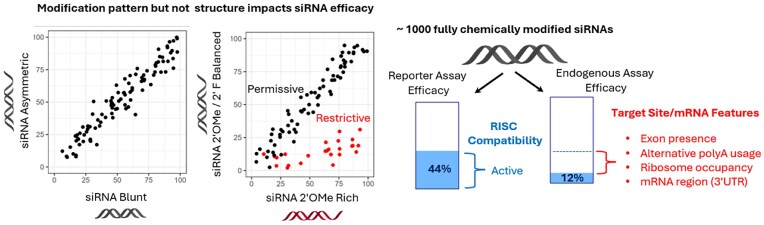

## Introduction

Chemically modified small interfering RNAs (siRNAs) are changing the world of medicine. siRNAs are 21- to 25-nucleotide (nt) double-stranded oligonucleotides that, upon cellular uptake, harness RNA interference (RNAi) to regulate gene expression. The antisense or “guide” strand of the siRNA is loaded into the RNA-induced silencing complex (RISC), which then searches for and cleaves complementary messenger RNA (mRNA) to prevent target protein expression. Because target specificity is determined simply by the guide strand sequence, siRNAs represent a highly programable molecular entity for selectively silencing a wide range of disease targets previously considered undruggable. Since 2018, six siRNA drugs have been approved for clinical use (patisiran, givosiran, lumasiran, inclisiran, and vutrisiran), with dozens of others in clinical development [1]. The clinical success of siRNA is dependent on efficient delivery to disease tissues.

The current siRNA delivery platform dominating the clinic is conjugate-mediated delivery by *N*-acetylgalactosamine (GalNac), which selectively delivers to liver [2]. To achieve systemic delivery to extrahepatic tissues, other conjugates are being investigated, including hydrophobic moieties [3, 4], antibodies [5, 6], and peptides [7]. Effective local delivery to extrahepatic tissues like lung [8] and brain [9] has been achieved by increasing compound size via multivalency. Regardless of the chemical conjugate used [10–12], full chemical modification is required [12] to stabilize siRNA in the harsh endosome environment following cell uptake. This stabilization is thought to define the long-term efficacy of siRNA drugs by creating an intracellular depot of siRNA that gets slowly released over time into cytoplasm for loading into RISC [13]. Nevertheless, chemical modifications can have a negative impact on RISC function [14].

Publicly available siRNA design algorithms have been developed from unmodified siRNA datasets, mainly generated from reporter assays, but are not predictive of fully chemically modified siRNA efficacy [14]. Some of the most common ribose modifications used to stabilize pre-clinical and clinical siRNAs are 2′-*O*-methyl (2′-OMe) or 2′-fluoro (2′-F) [15], which can be applied in different patterns along the length of the siRNA. How different 2′-OMe/2′-F patterns on siRNA affect RISC function and duration of effect [11] has been evaluated for only a few sequences, making it difficult to identify general design parameters across many sequences. Whether design rules defined by one modification pattern translate to another is also unknown.

siRNA duplex structure may also significantly impact efficacy [16]. Whereas naturally occurring siRNA duplexes have a 2-nt overhang at the 3′ end of the guide and passenger strand strands [17], creating an “asymmetric” structure, many different siRNA duplex structure designs are used in research. In the clinic, siRNA duplex structure design is largely driven by Intellectual Property (IP) and chemistry, manufacturing, and control (CMC) considerations. GalNAc-conjugated siRNA delivery to liver show potent silencing across different siRNA duplex structures [16]. Yet, siRNA duplex structure variably impacts efficacy in extrahepatic tissues [16]. In muscle, lung, and heart, siRNAs with a longer 5-nt guide strand overhang demonstrate better silencing than those with a 2-nt guide strand overhang, and both asymmetric structures outperform the blunt structure (i.e. no overhang). Yet, in fat tissue, blunt structure compounds achieve the best silencing [16]. The degree to which structure (asymmetric versus blunt) impacts efficacy *in vivo* and *in vitro* appears to be sequence-dependent [12, 16, 18]. Large-scale analysis is needed to define siRNA sequence features that work well in one structure over the other.

Complicating efforts to identify siRNA parameters impacting efficacy is the use of different assays to measure siRNA efficacy in cells. A reporter construct, wherein part of the gene or just the targeting site is positioned in the 3′-UTR of a luciferace is widely used [19]. This setup enables cost-effective and simple evaluation of the ability of chemically modified siRNA to load into RISC and cleave the mRNA target region in an ideal, isolated context. The translatability of efficacy data from reporter assays to data from native mRNA assays is unclear. In some cases, the assays correlate well [20], but multiple exceptions have been reported [8], suggesting the native context of the target mRNA impacts siRNA efficacy. For example, Alterman *et al.* [21] observed low efficacy for an siRNA targeting the 3′ untranslated region (UTR) of the *HTT* gene because the primary isoform expressed in the cell line used for screening did not include this region. Currently, therapeutic lead identification requires extensive, laborious screening in a native mRNA context. Delineating the efficacy impacts of siRNA parameters and native mRNA target/model system factors may help to lower the barrier for entry for academic labs planning to use chemically modified siRNAs in their research.

Here, we perform a systematic, unbiased evaluation of factors contributing to fully chemically modified siRNA efficacy. We designed a panel of siRNAs randomly distributed across the length of four neurodegenerative disease genes and evaluated the relative contributions of siRNA parameters (chemical modification pattern, structure, and base preference) and native context factors (mRNA target region selection, mRNA translational efficiency, polyadenylation site sequence selection, and exon usage) on efficacy. We demonstrate that this multiplicity of factors impacts siRNA efficacy and provide a framework of how to consider each during therapeutic siRNA design.

## Materials and methods

Data Availability/Sequence Data Resources: 3P-seq data available on geo; accession number: GSE231101

### siRNA design

We made selections from all possible 20-nucleotide targeting sequences for *APP* (NM_000484), *BACE1* (NM_012104), *MAPT* (NM_001123066), and *SNCA* (NM_000345). First, we excluded sequences with (i) ≥60% G-C content because it is known to negatively impact silencing [22], and (ii) CCCC or GGGG stretches due to synthetic limitations. To minimize off-target effects, we also excluded siRNAs wherein positions 2–17 of the guide strand sequence were found to be homologous to any other human genes. While this filtering step does not remove the possibility of off-target effects mediated through seed sequence complementarity, it will preclude any other transcripts from being sliced by Ago2 loaded with this siRNA. Next, we generated lists from the remaining sequences for each target. Each list contained every 50th remaining sequence and all sequences containing the polyadenylation site sequence “AAUAAA”. We selected 48 sequences from each list by including 5 sequences containing the polyadenylation site sequence, and then selecting 43 sequences from the open reading frame (ORF) (6–26 sequences) or the 3′ UTR (17–37 sequences) depending on availability in the remaining lists for each target.

In the field, it is widely recognized that a common strategy for identifying functional siRNAs involves conducting a secondary walk around the primary hits identified in the initial screen. To quantitatively evaluate the relative contribution of this approach, we designed a second set of siRNAs based on the “walk around the primary hit” concept. For the second set of siRNAs (i.e. “Walk Around Hits”), we selected new sequences whose start sites (i.e. the first nucleotide at 5′ end of the 20-mer target region) were within 10 nucleotides upstream or downstream of the start site of each sequence that demonstrated efficacy in the first set of screens. Effective sequences were defined as those resulting in ≤ 40% mRNA expression in the first set of QuantiGene screens in any chemical modification scaffold tested. Note that this includes additional scaffolds not included in this manuscript (see Supplementary Table S3 for these results). We then spaced the sequence start sites two nucleotides apart and filtered these lists as described above.

For the third set of siRNAs (i.e. “3′ UTR Selective”), we designed sequences targeting the regions of *MAPT* and *SNCA* 3′ UTRs before the first polyadenylation site sequence. To do this, we made selections from all possible 20-nucleotide targeting sequences in these regions, filtered these lists as above, and then selected every other sequence.

### Oligonucleotide synthesis

Oligonucleotides were synthesized by phosphoramidite solid-phase synthesis on a Dr. Oligo 48 (Biolytic, Fremont, CA), using modified protocols. Modified 2′-F, 2′-OMe phosphoramidites with standard protecting groups were used. Bis-cyanoethyl-*N,N*-diisopropyl (CED) phosphoramidite was used for the addition of the 5′-phosphate. All phosphoramidites were purchased from Chemgenes, Wilmington, MA. Phosphoramidites were dissolved at 0.1 M in anhydrous acetonitrile (ACN), with added anhydrous 15% dimethylformamide in the 2′-OMe-uridine amidite. 5-(Benzylthio)-1H-tetrazole (BTT) was used as the activator at 0.25 M. Detritylations were performed using 3% trichloroacetic acid in dichloromethane. Capping reagents used were CAP A (20% *n*-methylimidazole in can) and CAP B (20% acetic anhydride and 30% 2,6-lutidine in ACN). Reagents for capping and detritylation were purchased from American International Chemical (AIC), Westborough, MA. Phosphite oxidation to convert to phosphate or phosphorothioate was performed with 0.05 M iodine in pyridine-H_2_O (9:1, v/v) purchased from AIC or 0.1 M solution of 3-[(dimethylaminomethylene)amino]-3*H*-1,2,4-dithiazole-5-thione (DDTT) in pyridine purchased from ChemGenes for 4 min. Phosphoramidite coupling times were 4 min. Unconjugated oligonucleotides were synthesized on 500 Å long-chain alkyl amine (LCAA) controlled pore glass (CPG) functionalized with Unylinker terminus (Chemgenes). Cholesterol conjugated oligonucleotides were synthesized on a 500 Å LCAA-CPG support, where the cholesterol moiety is bound to tetra-ethylene glycol through a succinate linker (Chemgenes). Syntheses were done on a 1-μmol scale. Synthesis columns were custom-packed at LGC Genomics, Alexandria, MN.

### Deprotection and purification of oligonucleotides for sequence screening

Synthesis columns containing the oligonucleotides covalently attached to the solid supports were rinsed with 50 μl of Milli-Q water and then cleaved and deprotected at 65°C, 40 psi for 1 h with Ammonia gas (Airgas Specialty Products) in a reaction chamber. Columns with cleaved and deprotected oligonucleotides were washed with 1 ml of 0.1 M sodium acetate in 85% ethanol aqueous solution, followed by rinse with an 85% ethanol aqueous solution. The excess ethanol was dried from the column on a vacuum manifold. Finally, the oligonucleotides were eluted off the columns with MilliQ water.

### LC–MS analysis of oligonucleotides

The identity of oligonucleotides is verified by LC–MS analysis on an Agilent 6530 accurate mass Q-TOF using the following conditions: buffer A: 100 mM 1,1,1,3,3,3-hexafluoroisopropanol (HFIP) (Oakwood Chemicals) and 9 mM triethylamine (TEA) (Fisher Scientific) in LC–MS grade water (Fisher Scientific); buffer B: 100 mM HFIP and 9 mM TEA in LC–MS grade methanol (Fisher Scientific); column, Agilent AdvanceBio oligonucleotides C18; linear gradient 0–35% B 5 min was used for unconjugated oligonucleotides; linear gradient 50%–100% B 5 min was used for cholesterol conjugated oligonucleotides; temperature: 60°C; flow rate: 0.85 ml/min. LC peaks are monitored at 260 nm. MS parameters: Source, electrospray ionization; ion polarity, negative mode; range, 100–3 200 *m/z*; scan rate, 2 spectra/s; capillary voltage, 4000; fragmentor, 200 V; gas temperature: 325°C.

### RNAscope FISH

RNAscope probe sets for human *APP*, *BACE1*, *MAPT*, *SNCA*, and *HPRT* were obtained from ACDBio (#418321; #422541; #408991; #605681; #310341-C2). RNAscope, using the RNAscope Fluorescent Multiplex Kit (ACDBio; #320850), was performed following the manufacturer’s instructions.

### Imaging

Images for quantification (results shown in Fig. 10) were acquired with a Leica DMi8 inverted microscope (Leica Microsystems) with an HC PL Apochromatic (APO) 63×/1.40 oil-immersion objective and a Hamamatsu C11440 ORCA-Flash 4.0 camera. Z stacks were acquired in three different channels. Images within the same panel were acquired under the same light intensity settings and exposure times set individually for each channel. Images were processed using LAS X.

Representative images shown in Fig. 10 were acquired with a Leica SP8 LIGHTNING laser scanning confocal microscope equipped with an HC PL APO CS2 63×/1.40 oil-immersion objective, and OPSL 488, OPSL 552, and Diode 638 laser lines were used to acquire images on the manufacturer’s LAS X 3.5.5.19976 software. A PMT detector was used for the 552 channel and HyD detectors were used for all other channels. Each channel was acquired sequentially with excitation lasers (at 1% intensity for all channels) and emission ranges as follows (all in nm): 488 (493–549), 552 (557–659), and 638 (775–780). All images acquired on this microscope were used for qualitative assessment and never quantitatively analyzed.

### Image analysis

Image processing of RNAscope images was performed as described previously [23].

### Cell culture (HeLa, SH-SY5Y, U87, RAG, and 3T3 cells)

HeLa cells (ATCC, #CCL-2), U87 cells (ATCC HTB-14), RAG cells (ATCC CRL-142), and 3T3 cells (ATCC CL-173) were maintained in Dulbecco’s Modified Eagle’s Medium (DMEM) (Cellgro, #10-013CV), and SH-SY5Y cells (ATCC CRL-2266) were maintained in DMEM/F-12 (Gibco, #10565-018). The media was supplemented with 9% fetal bovine serum (FBS) (Gibco, #26140) and all cells were grown at 37°C and 5% CO_2_. Cells were split every 2 to 7 days and discarded after 15 passages.

### Transfection of APP, BACE1, MAPT, and SNCA target sequences into HeLa cells for dual-glo luciferase assay

Complementary DNA (cDNA) sequences corresponding to 20-nt long, unique regions of target mRNAs (see Supplementary Table S4) were cloned into psiCHECK-2 vectors (Promega, Madison, WI; C8021) according to the manufacturer’s protocol. HeLa cells were plated (3^6^ or 6^6^ cells/10-cm dish) and grown overnight (O/N) to ∼80% confluency before being transfected with 12 μg of psiCHECK-2 plasmid using Lipofectamine 2000 (Invitrogen, Carlsbad, CA; 11668019) according to the manufacturer’s protocol, which recommends >90% cell confluency at the time of transfection and plasmid DNA (μg) and Lipofectamine 2000 (μl) concentration ratios between 1:0.05 and 1:5. We found that 12 or 24 μg of plasmid: 60 μl of Lipofectamine 2000 (i.e. 1:5 or 1:2.5 ratio) produced appropriate signal-to-noise levels (>10) for all plasmids. Six hours after transfection, the media was changed to reduce cell death. Transfected cells were grown O/N before transfer and treatment with siRNAs.

### Direct delivery (i.e. passive uptake) of oligonucleotides

Transfected HeLa cells were diluted to 8000–10 000 cells/50 μl DMEM containing 6% FBS; U87, RAG, and 3T3 cells were diluted to 8000–10 000 cells/50 μl DMEM containing 6% FBS; SH-SY5Y cells were diluted to 7500 cells/50 μl DMEM/F-12 containing 6% FBS.

siRNAs were diluted to twice the maximum test concentration in OptiMEM (Carlsbad, CA; 31985-088) and a subset of compounds were serially diluted to create 7-point dose responses. Fifty microliters of diluted siRNA was added to 50 μl of diluted cells in 96-well cell culture treated plates, resulting in a final concentration of 1.5 μM siRNA (*APP*, *BACE1*, *MAPT*, *SNCA*, *SLC5A2*, *MECP2*, and *HTT* targeting compounds) or 0.5 μM siRNA (*Furin* and *Ace2* targeting compounds) for the maximum dose and 3% FBS. Cells were incubated for 72 h at 37°C and 5% CO_2_.

### Methods for quantitative analysis of target expression

Reporter assay: Renilla luciferase signal was used as a proxy for target mRNA knockdown and quantified using the Dual-Glo^®^ Luciferase Assay System, according to the manufacturer’s protocol (Promega, #E2940). Luminescence was detected on a Veritas Luminometer (Promega, #998-9100) or a Tecan M1000 (Tecan, Morrisville, NC). The mean results from blank control wells (i.e. cells without transfected plasmid) were subtracted from sample wells and used to ensure inter-assay linearity. For each cell treatment plate, Firefly luciferase was used to monitor plasmid transfection and cell death and used as a normalization control. Data were then plotted as a percentage of the mean results from untreated cells. Positive control (*HTT*-targeting; HTT_10 150 compound information in Supplementary Table S6) and negative control (nontargeting) siRNAs were used in each experiment.

Native assay: mRNA was quantified using the QuantiGene 2.0 assay kit (Affymetrix, QS0011). Cells were lysed in 250 μl of diluted lysis mixture composed of 1-part lysis mixture (Affymetrix, 13228), two parts of H_2_O, and 0.167 μg/μl proteinase K (Affymetrix, QS0103), for 30 min at 55°C. Probe sets for human *APP*, *BACE1, MAPT, SNCA*, *HPRT*, *ATF5*, *HTT*, *STAT3*, and *SOD1* (Affymetrix SA-10693, SA-16732, SA-15486, SA-50528, SA-10030, SA-16699, SA-50339, SA-50595, and SA-10232) and mouse *STAT3*, *VIM*, and *HPRT* (Affymetrix SB-26535, SB-15089, and SA-325390) were diluted and used according to the manufacturer’s recommended protocol. Cell lysates were mixed thoroughly before 20–80 μl of lysate and 20 μl of probe set mixture were added to each well of a capture plate in triplicate. 0–60 μl of H_2_O was also added, such that each well contained 100 μl total. The amount of lysate used was validated to be within the linear range of the assay and produce appropriate signal-to-noise (>5; see Supplementary Fig. S1). Chemiluminescence was detected on a Veritas Luminometer (Promega, #998-9100) or a Tecan M1000 (Tecan, Morrisville, NC). The mean results from blank control wells (i.e. cells without probe set) were subtracted from sample wells and used to ensure inter-assay linearity. For each cell treatment plate, *HPRT* was used to monitor cell death and as a normalization control. Data were then plotted as a percentage of the mean results from untreated cells. Positive control (*HTT*-targeting; HTT_10150 compound information in Supplementary Table S6) and negative control (nontargeting) siRNAs were used in each experiment.

### Quality control, statistical analyses, and exclusion of data for single point efficacy data

siRNA duplex formation was verified by running every single duplex synthesized for this study on 20% TBE gels (Novex, EC63155BOX). If a duplex did not form properly, it was removed from analysis.

Seven of the *APP*-targeting siRNA sequences were inadvertently designed to target regions downstream of a poly-adenylation site (PAS) site, meaning the targeted region was not actually present in the expressed mRNAs. As a result, these compounds were excluded from the analysis. Additionally, reporter assay data for one *SNCA*-targeting siRNA were excluded because three of its targeted sequence bases overlapped with the positive control sequence at the end of the plasmid insert, potentially affecting the efficacy results.

Statistical outliers were identified using GraphPad Prism 9.0 software (GraphPad Software, Inc., San Diego, CA) Grubbs’ analysis with Alpha = 0.2, and were excluded from calculations of the average and standard deviation values. For ease of plotting, target expression values in Figs 1–8 and Figs 12 and 13 are capped at 100%, and standard deviations shown are calculated from capped values.

### Selection of siRNAs for evaluation by concentration response in Supplementary Fig. S4

For *APP*, selected compounds met the following criteria: (i) were most effective (i.e. resulted in ≤ 30% target mRNA expression and/or were within top 10% of compounds in each chemical modification scaffold and resulted in ≤40% target mRNA expression) or (ii) had the biggest efficacy changes across different chemical scaffolds (i.e. siRNAs that resulted in <60% target mRNA expression and resulted in ≥20% difference in target mRNA expression across different chemical modification scaffolds). For *MAPT*, the only three sequences that resulted in target mRNA expression ≤30% in Blunt 2′-OMe/-F or Asymmetric 2′-OMe/-F were selected.

### Statistical analyses for concentration response data

Dose response data were analyzed using GraphPad Prism 9.0 software (GraphPad Software, Inc., San Diego, CA). Average untreated (i.e. UNT) value (i.e. 100%) for entire test plate plotted and used for all calculations. Concentration-dependent IC_50_ curves fitted using the log(inhibitor) versus response–variable slope (four parameters) method. The lower limit of the curve was set to >0, and the upper limit of the curve was set to a constant = 100. AUCs calculated using automatic software settings, i.e. Baseline: Y = 0, Minimum Peak Height: ignore peaks <10% of the distance from *Y*_min_ to *Y*_max_. AUC calculations excluded the datapoint for untreated control results set to 100%. Statistical outliers for treated values were identified using ROUT analysis with *Q* = 1% and were excluded from the calculations of the fitted curve and AUC.

### 3P Sequencing and analysis

3P sequencing protocol was performed as described previously [24], with the following exceptions: after elution of the mRNA 3′ ends from oligo-d(T) beads, a mixture of two pre-adenylated 3′ DNA adapters (AppNN NGT ANN NTA GNN NGA TCG TCG GAC TGT AGA ACT CTG AAC /3AmMO/, AppNN NAC TNN NGA TNN NGA TCG TCG GAC TGT AGA ACT CTG AAC /3AmMO/), adenylated as described in Hildebrand *et al.* [25], were ligated to these eluted mRNAs using Rnl2KQ. The ligation products were gel purified and phosphorylated as described in Jan *et al.* [24], and a 5′ RNA adapter (rCrCrU rUrGrG rCrArC rCrCrG rArGrA rArUrU rCrCrA rNrNrN rCrUrA rNrNrN rGrArC rNrNrN) was ligated to the 5′ RNA ends using Rnl1. This ligation product was then reverse transcribed using Superscript 2 (ThermoFisher cat #: 18064022) with the primer sequence: GTT CAG AGT TCT ACA GTC CGA CGA TC. The RT product was then gel purified and the final libraries were amplified using AccuPrime *pfx* DNA polymerase (ThermoFisher cat#: 12344024) and the following primers: Forward Primer: AAT GAT ACG GCG ACC ACC GAG ATC TAC ACG TTC AGA GTT CTA CAG TCC GA and Reverse Primer: CAA GCA GAA GAC GGC ATA CGA GAT CGT GAT GTG ACT GGA GTT CCT TGG CAC CCG AGA ATT CCA. The final amplified libraries were gel extracted, quantified, and sequenced on a NextSeq 550 (Illumina) for 79 cycles.

Data were pre-processed using the fastx_toolkit/0.014 to trim the adapter sequences from the 5′ and 3′ ends of reads, and de-duplicate reads. Then, the reverse complement of the pre-processed data was aligned to the hg38 reference genome using STAR/2.7.6a. The resulting .bam file was filtered to include only *APP*, *BACE1*, *MAPT*, or *SNCA* by location: chr21:25878550–26173128, chr11:117283686–117318256, chr17:45892382–46030334, and chr4:89722099–89840304, respectively.

Samtools/1.9 was used to convert these filtered .bam files to .sam files, and these .sam files were evaluated similarly to Spies *et al.* [26] Briefly, tags representing ≥10% tag reads were defined as a poly(A) site, tags within 20-nt windows were consolidated into one poly(A) site, and each poly(A) site was required to include ≥1 tag with ≥4 terminal 3′ A’s. Sites that meet these requirements are marked with black arrows in Fig. 11.

Sorted .bam files were supplied as input to generate raw per base count .wig files from gene of interest coordinates using IGVtools count (IGVTools/2.3.31). Normalized counts plotted in Fig. 11 were calculated by dividing the per base coverage by the total number of reads (i.e. the sum of the number of uniquely mapped reads and number of reads mapped to multiple loci divided by 10^6^).

### Analysis of RNA-sequencing data

FastQ files (SRR2932227 and SRR2932228, paired end) from Li *et al.* [27] were downloaded from the European Nucleotide Archive (accession number: PRJNA301726). Data were mapped to the hg38 reference genome that was filtered to include only NM accession numbers using STAR/2.7.6a. Resulting .bam files were then sorted using Samtools sort (Samtools/1.9). Sorted .bam files were supplied as input to generate raw per base count .wig files from gene of interest coordinates using IGVtools count (IGVTools/2.3.31). Normalized counts plotted in Figs 11 and 13 were calculated by dividing the per base coverage (sum of replicates 1 and 2) by the total number of reads for both replicates (i.e. the sum of the number of uniquely mapped reads and number of reads mapped to multiple loci for replicates 1 and 2 divided by 10^6^). TPM was quantified using RSEM/1.3.3 and used to determine percentage of reads aligned to each mRNA isoform and to calculate ribosome density.

### Analysis of ribosome profiling data

FastQ files (SRR15403639, single end) from Douka *et al.* [28] were downloaded from the European Nucleotide Archive (accession number: PRJNA753469). Data were pre-processed by using the fastx_toolkit/0.014 to trim the adapter sequence “AGATCGGAAGAGCACACGTCT” from the 3′ end of each read and filter the trimmed reads so that 90% of each read passed a quality threshold Phred score of 20. Then rRNA (FASTA file from RNAcentral) and tRNA (FASTA file from GtRNAdb at UCSC) reads were removed by performing bowtie2/2.4.1 alignment to each FASTA file and consecutively carrying forward the unaligned reads for analysis. Finally, one base was removed from the 3′ end of each read using cutadapt/2.9. The pre-processed data were aligned to the hg38 reference genome that was filtered to include only NM accession numbers using bowtie2/2.4.1. Resulting .bam files were then sorted using Samtools sort (Samtools/1.9). Sorted .bam files were supplied as input to generate raw per base count .wig files from gene of interest coordinates using IGVtools count (IGVTools/2.3.31). Normalized counts plotted in Fig. 13 and Supplementary Fig. S6 were calculated by dividing the per base coverage by the total number of reads (i.e. the sum of the number of uniquely mapped reads and number of reads mapped to multiple loci divided by 10^6^). TPM was quantified using RSEM/1.3.3 and used to calculate ribosome density.

### Assignment of mRNA positions and regions

For each gene, “mRNA positions” were defined using a mRNA “transcript” representing all mRNA isoforms expressed in undifferentiated SH-SY5Y cells according to RNA-seq and 3P-seq data analyses. This mRNA “transcript” was created by (i) filtering the hg38 reference genome (which had first been filtered to include only NM accession numbers) for relevant isoforms (i.e. those to which RNA-seq reads aligned), (ii) finding unique start and end locations for exons in the .gtf (note that if there was overlap for 2 + exons, the most extreme locations for each were used), and (iii) using these start and end locations to search for sequences in those ranges in the FASTA file and stringing them together. The resulting mRNA “transcripts” are shown in Supplementary Table S5. A similar method was used to define mRNA regions (i.e. 5′ UTR, ORF, and 3′ UTR): unique start and end locations for each region were found in relevant isoforms in the .gtf file, and the most extreme locations for each were used.

### Assignment of 50mer common regions

The 50mer target region includes 15 nucleotides on either side of the 20mer siRNA target sequence as well as the siRNA target in the context of the plasmid sequence (see Supplementary Table S4) or native mRNA sequence. The native mRNA sequence was defined as the consensus sequence across mRNA isoforms expressed in SH-SY5Y cells (i.e. those to which RNA-seq reads aligned); positions that contained different nucleotides across mRNA isoforms were excluded from analyses and are denoted as “?” in Supplementary Table S1.

### Definition of siRNA hotspots

Every siRNA hit was identified (i.e. siRNAs resulting in ≤35% mRNA expression). If there was one or more hit(s) with start sites within 50 nucleotides, that region (i.e. including the region 15 nucleotides upstream of the start site for hit 1–35 nucleotides downstream of the start site for hit 2+) was considered a hot spot.

### Thermodynamic stability calculations

Thermodynamic stabilities were calculated using nearest neighbor rules defined in Zuber *et al.* [29] Briefly, Δ*G*°_37_ was assigned for each pair of nucleotides in the 20mer siRNA target sequence (i.e. 1–2, 2–3… 19–20), then the average Δ*G*°_37_ at each pair was calculated for each group of sequences (e.g. permissive or restrictive). ΔΔ*G*°_37_ for each pair was calculated by subtracting the average for one group from the average for the other group (see Figs 2E and 3E).

### Random forest (RF) analysis

Analysis performed similarly to as described in Monopoli *et al.* [30]

Efficacy threshold selection: Effective threshold – h_1_ – and ineffective threshold – h_2_ – were defined to include siRNAs with reported expression values: ≤35% and >55%, respectively. siRNAs with expression values >35% but ≤55% were classified as undefined.

Feature parameterization: For each siRNA sequence, the 50-nt target site of the target mRNA was extracted to generate feature vectors for training the model. Specifically, the sequences were encoded into basic binary features using the following protocol [31]. A’s were represented as [1,0,0,0], U’s were represented as [0,1,0,0], C’s were represented as [0,0,1,0], and G’s were represented as [0,0,0,1]. Question marks (placed at positions where a base differed across prominently expressed isoforms) were excluded from analysis. For each 50-nt sequence, arrays of the encoded bases were appended in the order they appear in the sequence to form the final 200-dimensional one-hot encoding feature vector that the ML method was trained on. Each feature vector contains each nucleotide at each position in the full 50-nt target site sequence as features. Thus, the model considers the nucleotides on a per-position basis. Feature vectors were labeled with previously described efficacy classifications (effective/ineffective/undefined).

Assessment protocol: A training set containing 85% of the data and holdout dataset containing 15% were randomly selected using the Scikit-Learn *train_test_split* method, which ensured unbiased random partitioning of data into desired proportions and enabled equal distribution of effective and ineffective siRNAs when changing both h_1_ and h_2_ thresholds. The training dataset was used in *K*-fold cross-validation, during which it was partitioned randomly into ten *K* groups using the Scikit-Learn *KFold* method, which ensured random and even partitioning of the data. During partitioning of *K* groups, dataset classification was considered to ensure test groups were balanced with approximately equal numbers of effective and ineffective siRNAs.

In addition to the holdout dataset, external datasets were used to validate the predictive algorithm. Supplementary Table S6 shows the external dataset containing reporter assay data, and Supplementary Table S7 shows the external dataset consisting of native assay data.

Model performance was assessed by comparing the adjusted areas under the PR curve (AUCPR_adj_) [30].

Machine learning model training: Using the feature vectors, the supervised learning models were trained using the *Random Forest Classifier* from the *Scikit-Learn* Python Package [32]. All RF models were trained with the following default parameters: 200 total trees with a maximum tree depth of three nodes, and at least one sample per leaf. Model performance was also evaluated solely on the training set during training. The linear classifier models were trained using a published method that leverages an *ad-hoc* function and the three activity classification groups [14].

## Results

### Design of experimental dataset to evaluate the impact of siRNA and target mRNA factors on chemically modified siRNA efficacy

To systematically evaluate the contributions of different factors to chemically modified siRNA efficacy, we designed and synthesized a panel of siRNAs targeting 192 sites (each 20-nt long) randomly distributed within four mRNAs that contribute to pathophysiological hallmarks of Alzheimer’s disease (AD): amyloid β precursor protein (*APP*), β-Secretase 1 (*BACE1*), microtubule-associated protein tau (*MAPT*), and α-synuclein (*SNCA*). These mRNAs are differentially regulated and exhibit varied expression levels, making them more representative of a broader range of gene behavior *in vivo* compared to the highly and ubiquitously expressed genes commonly used in large-scale analyses of siRNA efficacy. While certain tissues (e.g. muscle and liver) predominantly express a limited number of highly and ubiquitously expressed genes, in other tissues (e.g. brain, kidney, and testis), these genes constitute only a relatively small proportion of the transcriptome [33].

By selecting sequences spanning the full length of mRNA ORFs and 3′ UTRs, we sought to introduce minimal targeting bias. We only excluded sequences with certain features already known to negatively impact siRNA efficacy or synthesis (see “Materials and methods” section). In general, the start sites of siRNA target sequences are spaced at least 50 nt apart. However, the spacing of siRNA target sequences and the representation of targeting regions were ultimately dictated by the overall length of each target, and the lengths of each ORF and 3′ UTR.

**Figure 1.**
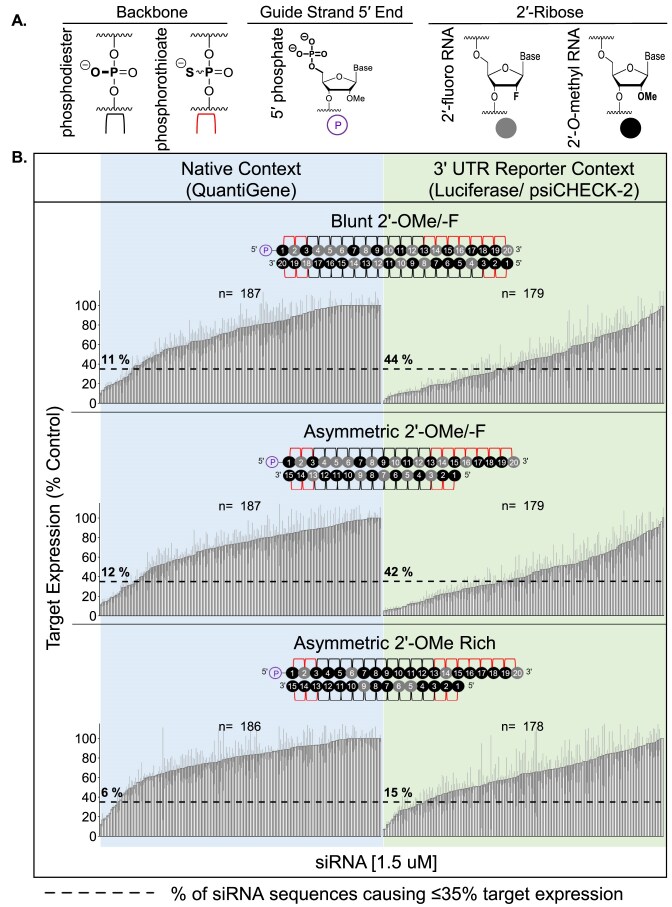
Experimental dataset demonstrates siRNA chemical pattern and assay impact hit rate. (**A**) siRNA chemical modifications used in this study. (**B**) siRNA target silencing results (*n* = 3, mean ± SD) in SH-SY5Y cells (native context, left panel) or 3′ UTR reporter context (HeLa cells, right panel) in three different chemical scaffolds used in study. The schematic of each chemical scaffold is shown above each set of results. SH-SY5Y or HeLa cells treated for 72 h. Target expression levels measured using the QuantiGene 2.0 RNA Assay (SH-SY5Y) or Dual-Glo^®^ Luciferase Assay System (HeLa) and calculated as percentage of untreated control.

The 192 siRNAs were synthesized in three different chemical modification pattern-duplex structure combinations (referred to as scaffolds) called Blunt 2′-OMe/-F, Asymmetric 2′-OMe/-F, and Asymmetric 2′-OMe Rich (Fig. 1). The first two scaffolds use relatively equal proportions of 2′-OMe and 2′-F modifications—the most common pattern in preclinical and clinical compounds [34]—in either a blunt or asymmetric (5-nt overhang) duplex structure. These duplex structures have shown the largest differences in efficacy in extrahepatic tissues *in vivo* [16]. The third scaffold is modified with 2′-OMe (except at select positions where 2′-OMe is known to negatively impact efficacy [11]) in the asymmetric (5-nt overhang) structure. For some sequences, this 2′-OMe-rich pattern maintains silencing efficacy *in vitro* and enhances tissue accumulation, retention, and potency *in vivo* [11, 18]. The effect of enhanced 2′-OMe content on siRNA efficacy has not been tested on a large number of sequences.

All scaffolds include a 5′ phosphate, which is present in naturally occurring siRNAs and is important for RISC loading [35], and a 2′-F modification at guide strand position 20 because it affords higher activity than 2′-OMe at this position [36]. In addition, phosphorothioate (PS) linkages replace phosphodiester bonds at all terminal nucleotides (including the 3′ single-stranded overhang in the asymmetric structure) to enhance nuclease resistance and promote cellular uptake [37]. Guide strands ranging from 19 to 25 nts paired to passenger strands at least 12–13 nt long can load into RISC [18, 38, 39]. Here, all scaffolds have 20-nt guide strands. Asymmetric compounds have 15-nt passenger strands, and blunt compounds have 20-nt passenger strands. These designs allowed us to evaluate the impact of duplex structure on siRNA efficacy without introducing potential variability induced by changing guide strand length.

The guide strand of the 2′-OMe/-F pattern maintains a balance of 2′-OMe and 2′-F like the standard, alternating 2′-OMe/-F modification pattern described by Allerson *et al.*, [34] with one exception: 2′-F modification at guide strand position 5 to increase target affinity in the seed (i.e. guide strand positions 2–8, responsible for initial binding and recognition of the target) [40], which is balanced by 2′-OMe modification at guide strand position 18, where the inclusion of 2′-F decreases siRNA activity [11]. The guide strand of the 2′-OMe rich pattern is modified with 2′-OMe except at positions 2, 6, 14, and 20, where 2′-OMe negatively impacts efficacy [11, 36].

Compared to 2′-OMe, 2′-F is not bulky and affords better base-stacking interactions [41, 42], which may increase duplex stability and inhibit passenger strand release to impair active RISC formation. Therefore, passenger strands in all scaffolds contain 2′-OMe across from 2′-F modifications and at terminal positions to promote duplex unwinding and passenger strand release [43]. This is particularly important in the Blunt scaffold due to the higher thermodynamic stability of the duplex. Because the passenger strand is longer in the Blunt scaffold than the Asymmetric 2′-OMe/ -F scaffold, it contains slightly higher 2′-OMe content overall. The 15-nt passenger strand of the Asymmetric 2′-OMe Rich scaffold contains 2′-OMe at all positions except 5, 6, and 9.

All siRNAs were conjugated to cholesterol to enable passive delivery to cells for efficacy evaluation. Although cholesterol can be cytotoxic at high doses *in vivo* [21, 44, 45], it is preferred for *in vitro* work as it is readily intercalated into the cellular membrane for efficient cellular internalization by endosomes without the need for transfection reagents [46].

### Design and validation of assays to measure siRNA efficacy in cells

The gene silencing efficacy of the siRNA panel was measured in SH-SY5Y cells using the QuantiGene 2.0 RNA assay (Fig. 1B, blue box) and in HeLa cells using a Dual-Glo^®^ Luciferase reporter assay, where the 20-nt siRNA target sites were cloned into the 3′ UTR of psiCHECK-2 reporter plasmids (see “Materials and methods” section) (Fig. 1B, green box). All sequences, chemical modification patterns, and corresponding efficacy results for the chemically modified siRNA panel are shown in Supplementary Table S1.

Using both assays allowed us to evaluate the ability of each siRNA to silence target mRNA expression in a native context (QuantiGene assay) and to load into RISC for target cleavage in an isolated context (Luciferase reporter assay). We were also able to systematically analyze how reporter assay data predict efficacy in a native context. Nevertheless, these two assays use different readouts for siRNA-mediated target silencing. Whereas the reporter assay readout depends on luciferase gene translation, the native assay readout relies directly on target mRNA levels. To ensure this did not confound results, we operated within the linear range of each assay (see “Materials and methods” section, Supplementary Fig. S1). We also performed a validation experiment for the reporter assay, measuring target mRNA silencing and reporter-based protein silencing. The mRNA and protein silencing results were similar (Supplementary Fig. S2).

SH-SY5Y cells (i.e. thrice sub-clones of the neuroblastoma cell line SK-N-SH derived from a metastatic bone tumor; ATCC CRL-2266) were used for native screening because they express all four mRNA targets and are a commonly used model system for neurodegenerative disorders. SH-SY5Y cells are not amenable to Lipofectamine 2000 [47], the reagent used to transfect psiCHECK-2 plasmid in the reporter assay, but we avoided significant disparities in siRNA delivery between cell lines caused by differing transfection efficiencies because siRNAs were delivered to each cell line passively via cholesterol-mediated uptake. Indeed, passive delivery of cholesterol-conjugated siRNAs resulted in similar target mRNA expression profiles (QuantiGene 2.0 RNA assay) in SH-SY5Y and HeLa cells (with AUC fold-changes ≤ 1.7) (Supplementary Fig. S3). This result suggests any difference in delivery had a minimal impact on efficacy results. The 1.7-fold difference in Ago2 levels between HeLa and SH-SY5Y cells (21.4 and 12.3 TPM, respectively, Human Protein Atlas) also has a minimal impact on efficacy. Thus, this experimental setup allowed for a systematic evaluation of the relative contributions of siRNA-specific features versus the native mRNA context to compound efficacy.

### Scaffold, modifications and sequence context alter siRNA efficacy

The siRNA panel efficacy results (Fig. 1B, average of three independent replicates) reveal two important trends. First, the hit rates (i.e. number of efficacious siRNA identified) for asymmetric and blunt scaffolds are generally similar in a native context (11% for Blunt 2′-OMe/-F versus 12% for Asymmetric 2′-OMe/-F) and in a reporter context (44% for Blunt 2′-OMe/-F versus 42% for Asymmetric 2′-OMe/-F), indicating that the relative contribution of duplex structure to *in vitro* efficacy is minimal. When comparing the 2′-OMe/-F and 2′-OMe Rich scaffolds, we see that some sequences are highly effective in both scaffolds, but the hit rates differ significantly in both a native context (12% for Asymmetric 2′-OMe/-F versus 6% for Asymmetric 2′-OMe Rich) and reporter context (42% for Asymmetric 2′-OMe/-F versus 15% for Asymmetric 2′-OMe Rich). The second trend we observe is that significantly more siRNAs induce silencing above a certain threshold (≤35% target expression, an inclusive but biologically meaningful [48] cutoff for effective sequences) in a reporter context versus the native context (44% versus 11% for Blunt 2′-OMe/-F; 42% versus 12% for Asymmetric 2′-OMe/-F; 15% versus 6% for Asymmetric 2′-OMe Rich). This result indicates that the native mRNA context negatively impacts siRNA efficacy, and not all siRNAs that efficiently enter the RISC complex and demonstrate activity in reporter assays are effective in silencing their target mRNA in a native environment.

To confirm native assay results, select siRNA compounds (*n* = 63; see “Materials and methods” section for selection criteria) were evaluated by a 7-point dose–response curve (Supplementary Fig. S4 and Supplementary Table S2). All siRNAs identified as efficacious in the primary screen exhibited high efficacy at the maximum treatment dose in a concentration-dependent manner (Supplementary Table S2, highlighted yellow). 86% of the time, compounds demonstrated a difference in target mRNA expression ≤20% across both assays. These data indicate that target silencing results obtained at a single treatment concentration in screens are reflective of outcomes across a broader treatment concentration range. This suggests that the experimental system produces consistent and robust results, enabling the analysis of broader trends. We next sought to better understand the two major features identified, starting with quantifying the impact of asymmetric versus blunt structure on siRNA hit rate.

### siRNA duplex structure has a limited impact on silencing efficacy and hit rate

**Figure 2.**
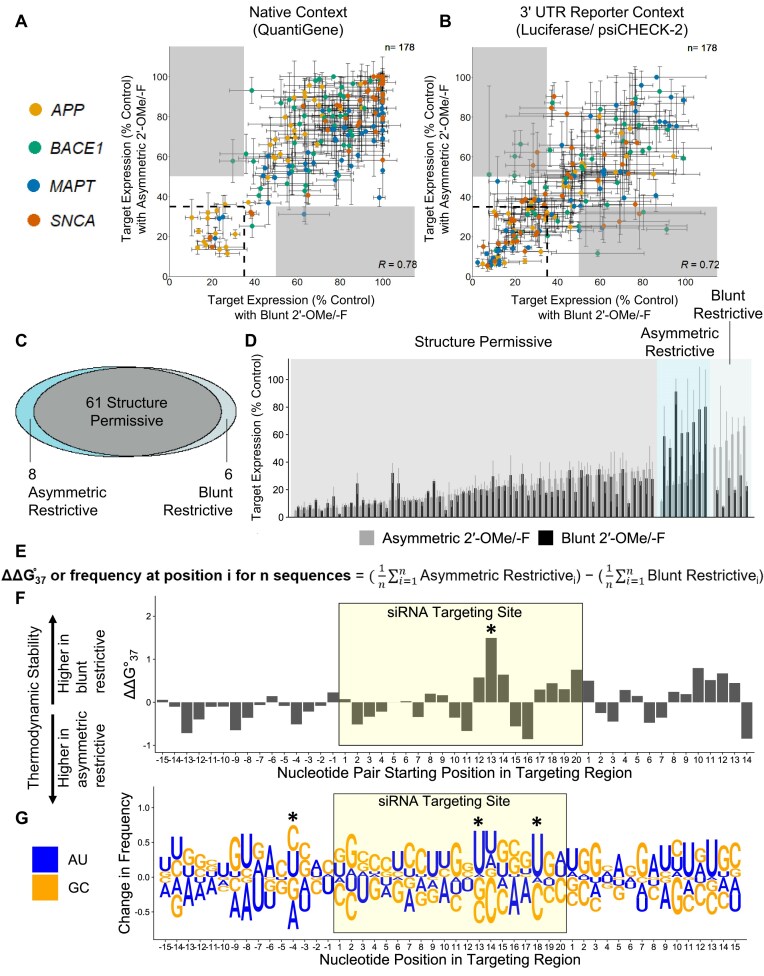
siRNA structure has limited impact on siRNA efficacy. (**A**, **B**, and**D**) siRNA target silencing results (*n* = 3, mean ± SD) in native context (A) or 3′ UTR reporter context (B) in Asymmetric 2′-OMe/-F (A and B: *y*-axis; D: gray bars) or Blunt 2′-OMe/-F scaffolds (A and B: *x*-axis; D: black bars). SH-SY5Y or HeLa cells treated for 72 h. Target expression levels measured using the QuantiGene 2.0 RNA Assay (SH-SY5Y) or Dual-Glo^®^ Luciferase Assay System (HeLa) and calculated as percentage of untreated control. Sequences causing ≤35% target expression in both scaffolds (i.e. permissive sequences) boxed by black dashed lines in (A) and (B) and shaded gray in (D). Sequences causing ≤35% target expression in one scaffold and >50% target expression in the other scaffold (i.e. restrictive sequences) shaded gray in (A) and (B) and shaded turquoise (asymmetric restrictive) or light blue (blunt restrictive) in (D). Pearson correlation coefficient displayed in bottom right corners in (A) and (B). (**C**) Proportional Venn diagram with numbers of structure permissive and restrictive siRNA sequences. (**E**) Equation used to calculate changes in thermodynamic stability (i.e. ΔΔ*G* °_37_) and frequency between asymmetric restrictive and blunt restrictive groups. *n* = number of sequences in each group, *i* = position in 50mer targeting region (**F**) ΔΔ*G* °_37_ plotted for each nucleotide pair in the 50mer targeting region, with each position number marking the first position of each nucleotide pair. *P*-values describe statistically significant differences between groups (*t*-test with Benjamini–Hochberg correction; *P < 0.05, nonsignificant differences unmarked). (**G**) Change in nucleotide frequency plotted for each nucleotide in the 50mer targeting region. *P*-values describe statistically significant differences between groups (Fisher’s exact test; *P < 0.05, nonsignificant differences unmarked).

Prior work suggests different siRNA duplex structures variably impact *in vitro* efficacy in a sequence-dependent manner [35, 40], but blunt siRNAs exhibit reduced efficacy *in vivo* [16], perhaps because longer passenger strands are more difficult to release for proper RISC function. In screening, it is also important to determine whether data generated using one chemical scaffold can be applied to another and whether the structure of an *in vitro*-identified hit can be engineered to better meet *in vivo* efficacy distribution requirements without comprimizing efficacy. In the present study, the efficacies of asymmetric 2′-OMe/-F and Blunt 2′-OMe/-F scaffolds were positively correlated in both assays (Pearson coefficient: Native = 0.78, Reporter = 0.72) (Fig. 2A and B). Most sequences effective in one scaffold showed similar efficacy in the other (i.e. ≤35% target mRNA expression, boxed with dashed lines in Fig. 2A and B; Fig. 2C and D, gray shaded regions); these sequences are defined as “permissive.” In the native mRNA context, only two sequences effective (i.e. ≤35% target mRNA expression) in one scaffold were ineffective (>50% target mRNA expression) in the other (Fig. 2A, shaded regions); these sequences are defined as “restrictive.” In a reporter context, six sequences were effective in the blunt but not asymmetric structure (i.e. blunt restrictive), and eight sequences were effective in asymmetric but not blunt structures (i.e. asymmetric restrictive, Fig. 2B, shaded regions; Fig. 2C and D, blue shaded regions). The threshold used to define ineffective sequences (i.e. >50% target expression) was selected to allow for a sufficient difference from effective sequences (i.e. ≤35% target expression) while remaining inclusive of the highest numbers of sequences for analysis. The 15% difference between 35% and 50% is greater than the average standard deviation for each assay (i.e. 11.5% in the 3′ UTR Reporter Context and 9.1% in the Native Context).

To explore what distinguishes blunt restrictive and asymmetric restrictive sequences, we compared the thermodynamic stabilities of each by calculating Δ*G*°_37_ for each position based on nearest neighbor rules [29] and looking at the change in Δ*G*°_37_ between the permissive and restrictive sequence groups (see “Materials and methods” section; equation displayed in Fig. 2E). Figure 2F plots this information, with each position marking the first position of each nt pair in the 50-nt targeting region.

### Chemical modification pattern has a significant impact on hit rate

**Figure 3.**
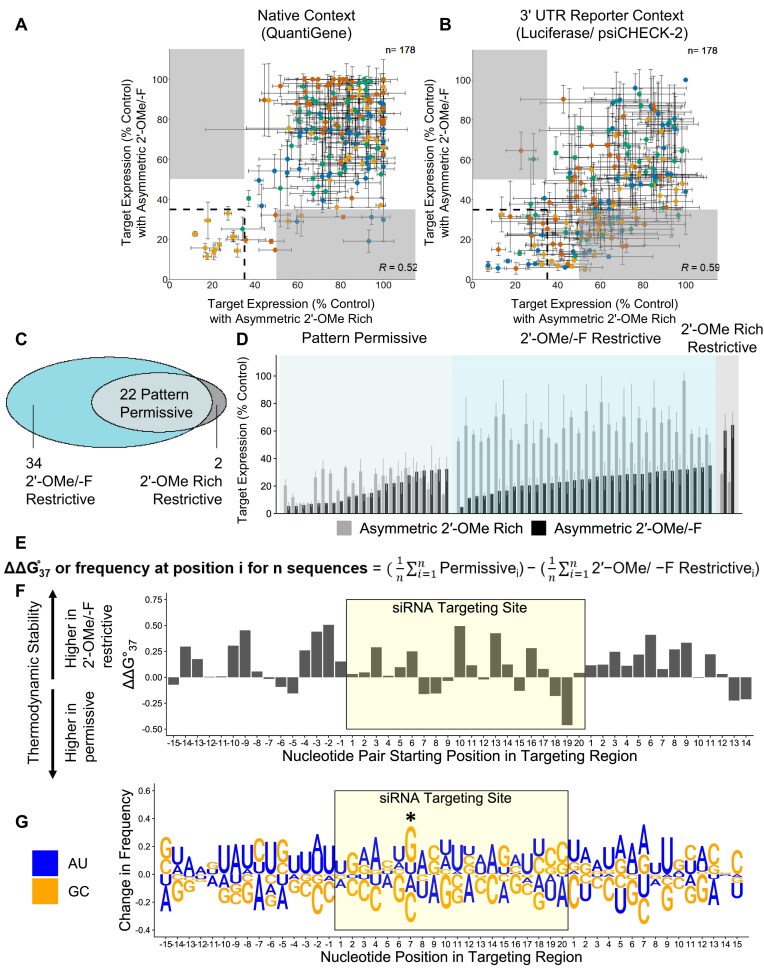
siRNA chemical pattern impacts siRNA efficacy. (**A**, **B**, **D**) siRNA target silencing results (*n* = 3, mean ± SD) in native context (A) or 3′ UTR reporter context (**B**) in Asymmetric 2′-OMe/-F (A and B: *y*-axis; D: gray bars) or Asymmetric 2′-OMe Rich scaffolds (A and B: *x*-axis; D: black bars). SH-SY5Y or HeLa cells treated for 72 h. Target expression levels measured using the QuantiGene 2.0 RNA Assay (SH-SY5Y) or Dual-Glo^®^ Luciferase Assay System (HeLa) and calculated as percentage of untreated control. Sequences causing ≤35% target expression in both scaffolds (i.e. permissive sequences) boxed by black dashed lines in (A) and (B) and shaded light blue in (D). Sequences causing ≤35% target expression in one scaffold and >50% target expression in the other scaffold (i.e. restrictive sequences) shaded gray in (A) and (B) and shaded turquoise (2′-OMe/-F restrictive) or gray (2′-OMe Rich restrictive) in (D). Pearson correlation coefficient displayed in bottom right corner in (A) and (B). (**C**) Proportional Venn diagram with numbers of structure permissive and restrictive siRNA sequences. (**E**) Equation used to calculate changes in thermodynamic stability (i.e. ΔΔ*G*°_37_) and frequency between pattern permissive and Asymmetric 2′-OMe/-F pattern restrictive groups. *n* = number of sequences in each group, i = position in 50mer targeting region (**F**) ΔΔ*G*°_37_ plotted for each nucleotide pair in the 50mer targeting region, with each position number marking the first position of each nucleotide pair. *P*-values describe statistically significant differences between groups (*t*-test with Benjamini–Hochberg correction; nonsignificant differences unmarked). (**G**) Change in nucleotide frequency plotted for each nucleotide in the 50mer targeting region. *P*-values describe statistically significant differences between groups (Fisher’s exact test; *P < 0.05, nonsignificant differences unmarked).

Next, we sought to better quantify the impact of 2′-OMe/-F versus 2′-OMe Rich chemical patterns on siRNA hit rate in our dataset. siRNA efficacies in the Asymmetric 2′-OMe/ -F and 2′-OMe Rich scaffolds are poorly correlated (Pearson coefficient: Native = 0.52, Reporter = 0.59) (Fig. 3A and B) because the 2′-OMe Rich pattern decreased efficacy for a large proportion of sequences.

In general, the most effective sequences are effective in both patterns (i.e. ≤35% target expression, boxed with dashed lines in Fig. 3A and B). For these permissive sequences, potency is not affected by changing the chemical pattern, as evidenced by similar half-maximal inhibitory concentration (IC_50_) and/or area under the curve (AUC) values across both scaffolds (Supplementary Fig. S4 and Supplementary Table S2). Nevertheless, in the reporter assay, which identified more effective sequences than the native mRNA (QuantiGene) assay, we did observe a number of differences between the two patterns, especially for more moderately effective sequences (target expression values closely approaching ≤35%). There are only two sequences effective in the 2′-OMe Rich scaffold that are ineffective in the 2′-OMe/-F scaffold (i.e. 2′-OMe Rich restrictive, Fig. 3C and D, gray shaded regions), but 34 sequences effective in the 2′-OMe/-F are ineffective in the 2′-OMe Rich scaffold (i.e. 2′-OMe/-F restrictive, Fig. 3C and D, turquoise shaded regions).

To explore what distinguishes the permissive sequences (Fig. 3C and D, light blue shaded regions) from 2′-OMe/-F restrictive sequences, we evaluated changes in thermodynamic stabilities and nt frequencies for each sequence group. We observed lower thermodynamic stability in the flanking regions surrounding the siRNA targeting site in permissive sequences (Fig. 3F). Looking at positional sequence preferences (Fig. 3G), we observed a trend towards having high-affinity seeds in permissive sequences, with a preference for C in positions 18–20 in the siRNA targeting region (positions 1–3 of the seed). However, the only individual base preference that reached statistical significance was G in position 7 of the siRNA targeting region (i.e. C in position 14 of guide strand). Increasing the threshold for ineffective sequences to 60% or 70% target expression results in the same trends (see Supplementary Fig. S5). Our findings identify sequence features that may facilitate efficient RISC interactions with a 2′-OMe-rich scaffold or that may characterize more effective target sites capable of tolerating less optimal chemical modification patterns. Consequently, it appears the modification pattern can have a significant and asymmetrical impact on efficacy. While the vast majority of compounds identified using a nonpermissive scaffold are likely to remain active in a permissive scaffold, the reverse is not true.

### siRNA hit rate is higher in reporter versus native contexts

**Figure 4.**
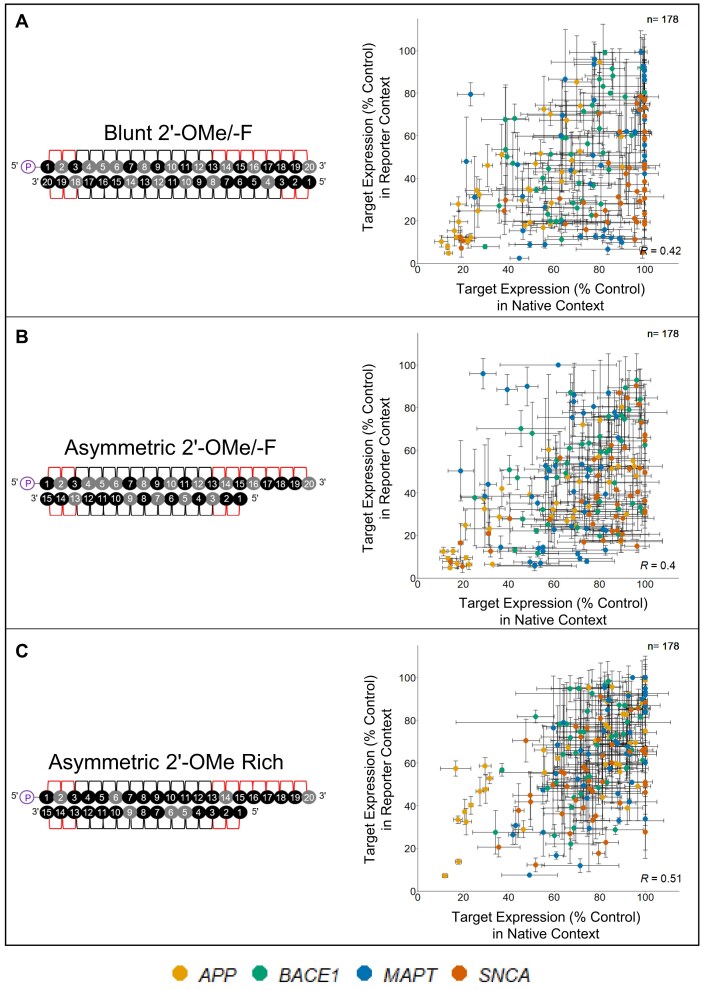
siRNA efficacy is higher in reporter versus native contexts. siRNA target silencing results (*n* = 3, mean ± SD) in native context (*x*-axis) or 3′ UTR reporter context (*y*-axis) in three different chemical scaffolds used in study: (**A**) Blunt 2′-OMe/-F bDNA, (**B**) Asymmetric 2′-OMe/-F, and (**C**) Asymmetric 2′-OMe Rich. The schematic of each chemical scaffold is shown next to the results. SH-SY5Y or HeLa cells treated for 72 h. Target expression levels measured using the QuantiGene 2.0 RNA Assay (SH-SY5Y) or Dual-Glo^®^ Luciferase Assay System (HeLa) and calculated as percentage of untreated control. Pearson correlation coefficient displayed in bottom right corner of each graph.

We next examined the overall correlation between efficacy data from the native mRNA versus reporter assays. The correlation is asymmetric: nearly every sequence that is effective in the native context is effective in the reporter context, but the reverse is not true (Fig. 4), indicating that there is a subset of sequences fully compatible with RISC entry and target recognition that fail to maintain efficacy in a native context. Thus, native context factors limit siRNA efficacy. This is consistent with previous reports showing siRNA-mediated silencing can be affected by intracellular localization and splicing of target mRNA [49]. However, the individual and combined impacts of native context factors on siRNA efficacy have not been wholly defined.

### Targets show different degrees of correlation between native and reporter contexts

**Figure 5.**
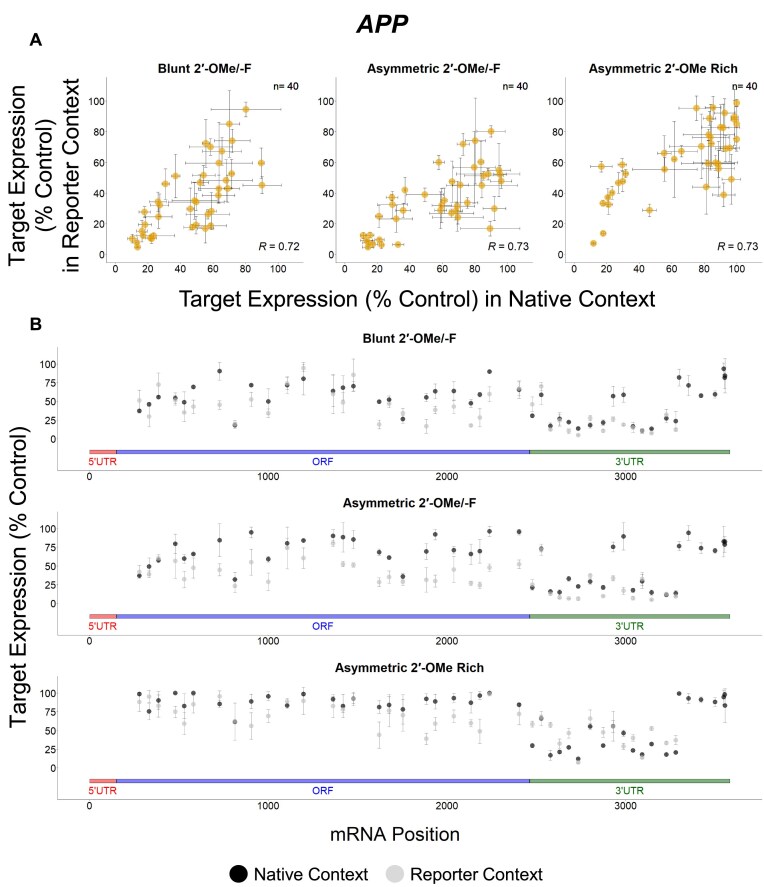
Degrees of correlation across native and reporter contexts for *APP* targeting compounds. siRNA target silencing results (*n* = 3, mean ± SD) in native context (**A**, *x*-axis; **B**, black datapoints) or 3′ UTR reporter context (A, *y*-axis; B, gray datapoints) in Blunt 2′-OMe/-F (A, left panel; B, top panel), Asymmetric 2′-OMe/-F (A, middle panel; B, middle panel), or Asymmetric 2′-OMe Rich scaffolds (A, right panel; B, bottom panel) for *APP*. SH-SY5Y or HeLa cells treated for 72 h. Target expression levels measured using the QuantiGene 2.0 RNA Assay (SH-SY5Y) or Dual-Glo^®^ Luciferase Assay System (HeLa) and calculated as percentage of untreated control. Pearson correlation coefficient displayed in bottom right corners in (A). Gene regions (labeled) are shown below graphs in (B).

**Figure 6.**
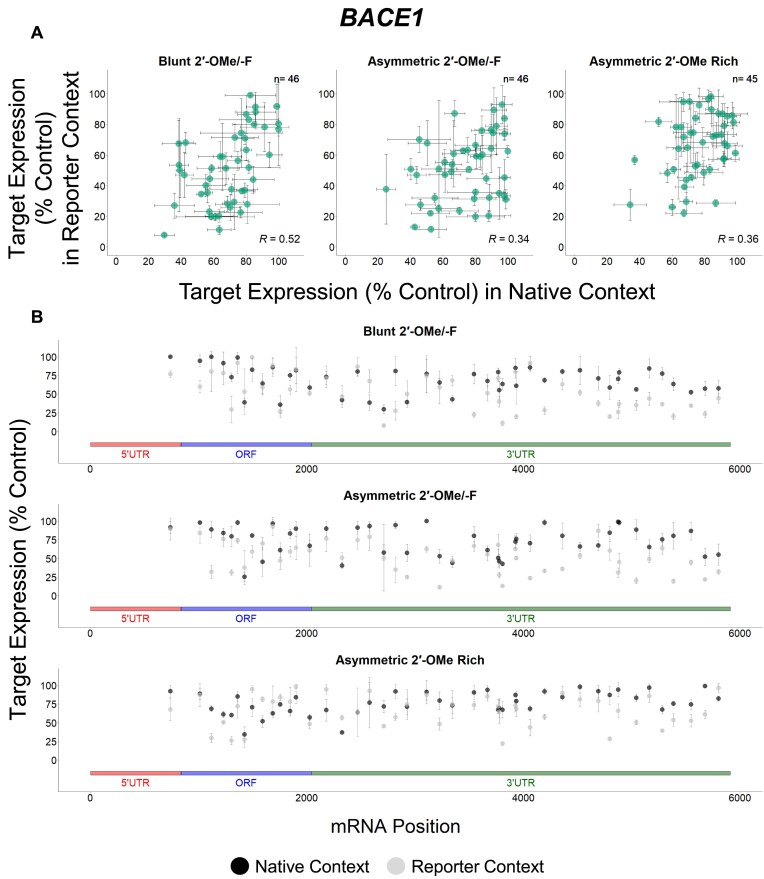
Degrees of correlation across native and reporter contexts for *BACE1* targeting compounds. siRNA target silencing results (*n* = 3, mean ± SD) in native context (**A**,*x*-axis; **B**, black datapoints) or 3′ UTR reporter context (A, *y*-axis; B, gray datapoints) in Blunt 2′-OMe/-F (A, left panel; B, top panel), Asymmetric 2′-OMe/-F (A, middle panel; B, middle panel), or Asymmetric 2′-OMe Rich scaffolds (A, right panel; B, bottom panel) for *BACE1*. SH-SY5Y or HeLa cells treated for 72 h. Target expression levels measured using the QuantiGene 2.0 RNA Assay (SH-SY5Y) or Dual-Glo^®^ Luciferase Assay System (HeLa) and calculated as percentage of untreated control. Pearson correlation coefficient displayed in bottom right corners in (A). Gene regions (labeled) are shown below graphs in (B).

**Figure 7.**
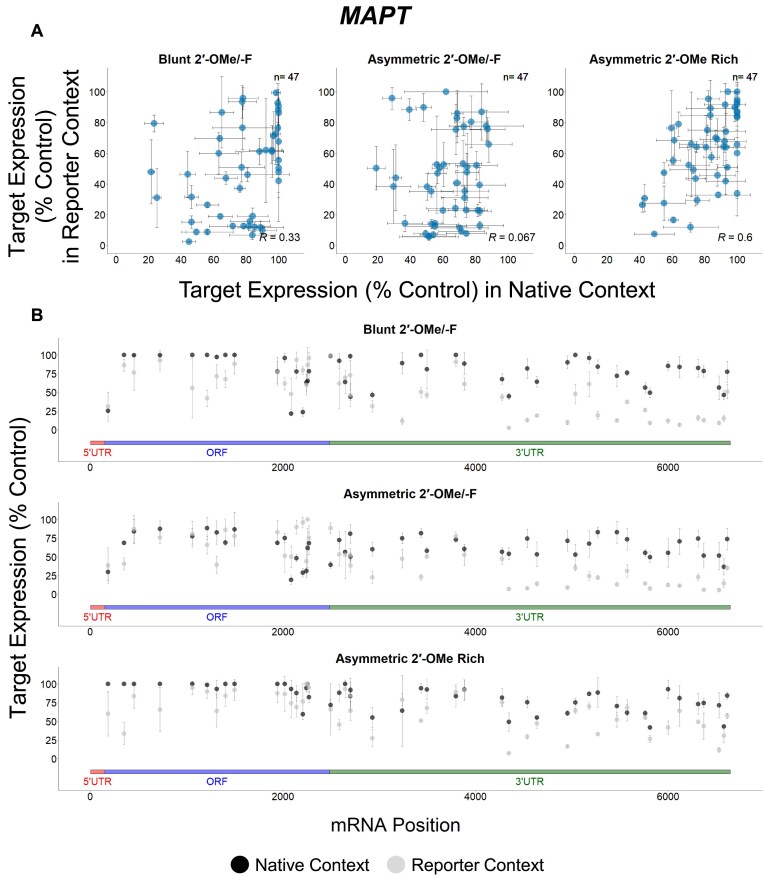
Degrees of correlation across native and reporter contexts for *MAPT* targeting compounds. siRNA target silencing results (*n* = 3, mean ± SD) in native context (**A**,*x*-axis; **B**, black datapoints) or 3′ UTR reporter context (A, *y*-axis; B, gray datapoints) in Blunt 2′-OMe/-F (A, left panel; B, top panel), Asymmetric 2′-OMe/-F (A, middle panel; B, middle panel), or Asymmetric 2′-OMe Rich scaffolds (A, right panel; B, bottom panel) for *MAPT*. SH-SY5Y or HeLa cells treated for 72 h. Target expression levels measured using the QuantiGene 2.0 RNA Assay (SH-SY5Y) or Dual-Glo^®^ Luciferase Assay System (HeLa) and calculated as percentage of untreated control. Pearson correlation coefficient displayed in bottom right corners in (A). Gene regions (labeled) are shown below graphs in (B).

**Figure 8.**
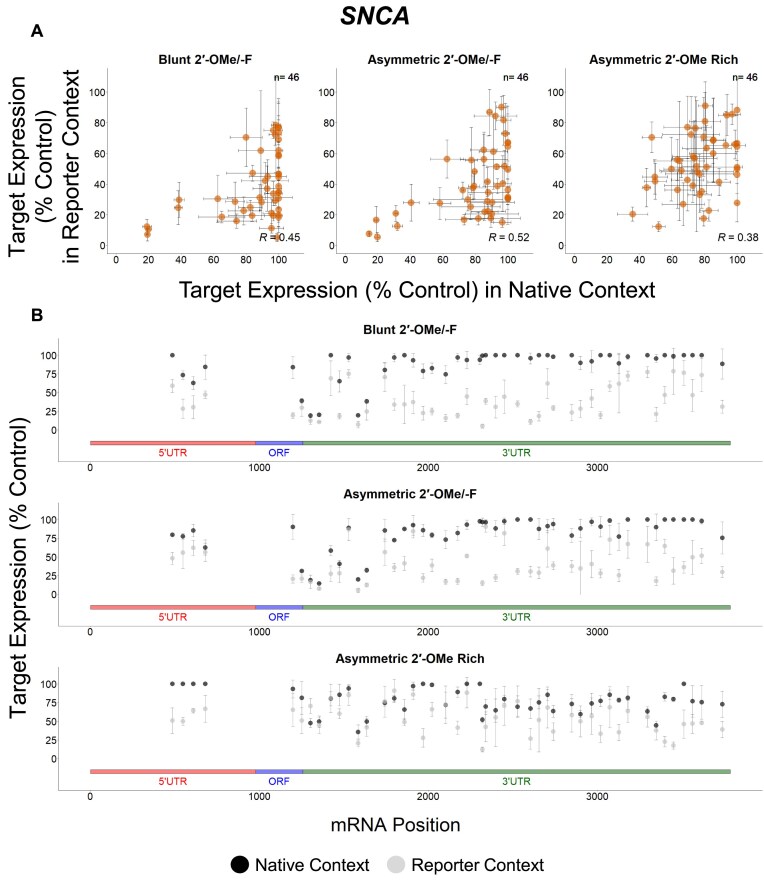
Degrees of correlation across native and reporter contexts for *SNCA* targeting compounds. siRNA target silencing results (*n* = 3, mean ± SD) in native context (**A**,*x*-axis; **B**, black datapoints) or 3′ UTR reporter context (A, *y*-axis; B, gray datapoints) in Blunt 2′-OMe/-F (A, left panel; B, top panel), Asymmetric 2′-OMe/-F (A, middle panel; B, middle panel), or Asymmetric 2′-OMe Rich scaffolds (A, right panel; B, bottom panel) for *SNCA*. SH-SY5Y or HeLa cells treated for 72 h. Target expression levels measured using the QuantiGene 2.0 RNA Assay (SH-SY5Y) or Dual-Glo^®^ Luciferase Assay System (HeLa) and calculated as percentage of untreated control. Pearson correlation coefficient displayed in bottom right corners in (A). Gene regions (labeled) are shown below graphs in (B).

To better understand the potential factors responsible for mRNA-specific features affecting siRNA efficacy, we analyzed the data for individual genes separately. Our data reveal that there are more *BACE1* (green), *MAPT* (blue), and *SNCA* (dark orange) targeting sequences exclusively effective in the reporter assay, while more *APP* (light orange) targeting sequences show efficacy in both assays (Figs 5–8). When the native and reporter data are plotted side-by-side for each target (Figs 5–8), *APP* shows a high degree of correlation across both assays (*R* ∼0.7 for all scaffolds), while correlations for the other targets are lower (*R* ∼0.3 for *BACE1*, *R* ∼0.07 for *MAPT*, and *R* ∼0.5 for *SNCA* for the asymmetric dataset). In addition, efficacy discrepancies between assays are observed in the 3′ UTRs for *BACE1*, *MAPT*, and *SNCA*, suggesting lower degrees of correlation are specific to certain regions within the target (Figs 5–8).

### siRNA efficacy is not limited by nuclear localization of the four targets in SH-SY5Y cells

**Figure 9.**
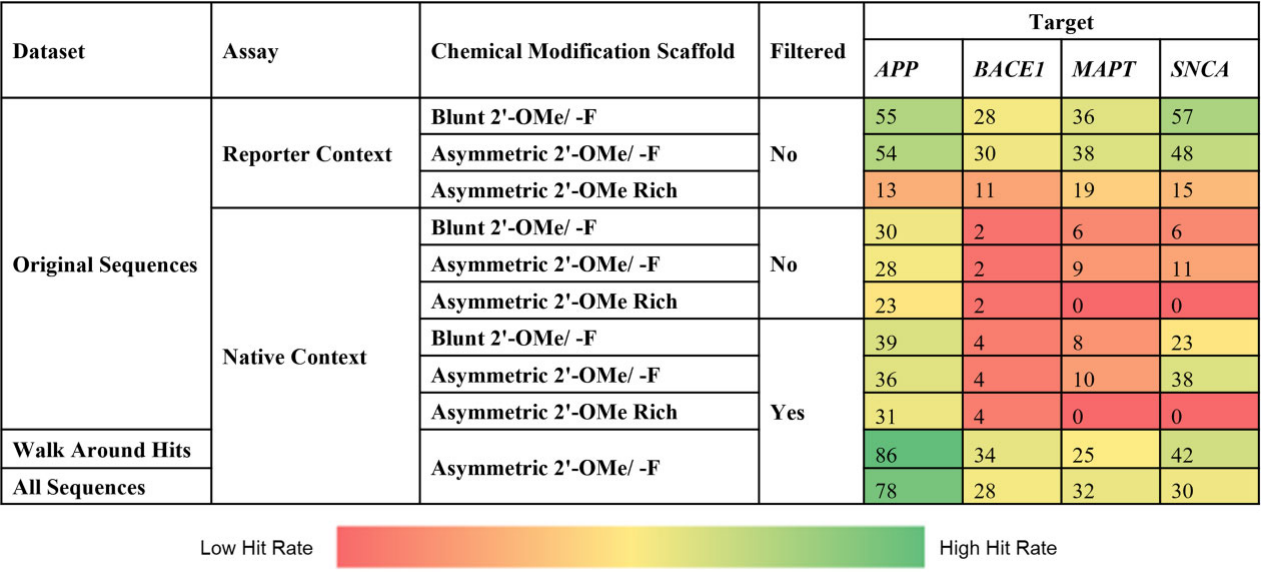
Percent of siRNAs with target expression ≤35%.

**Figure 10.**
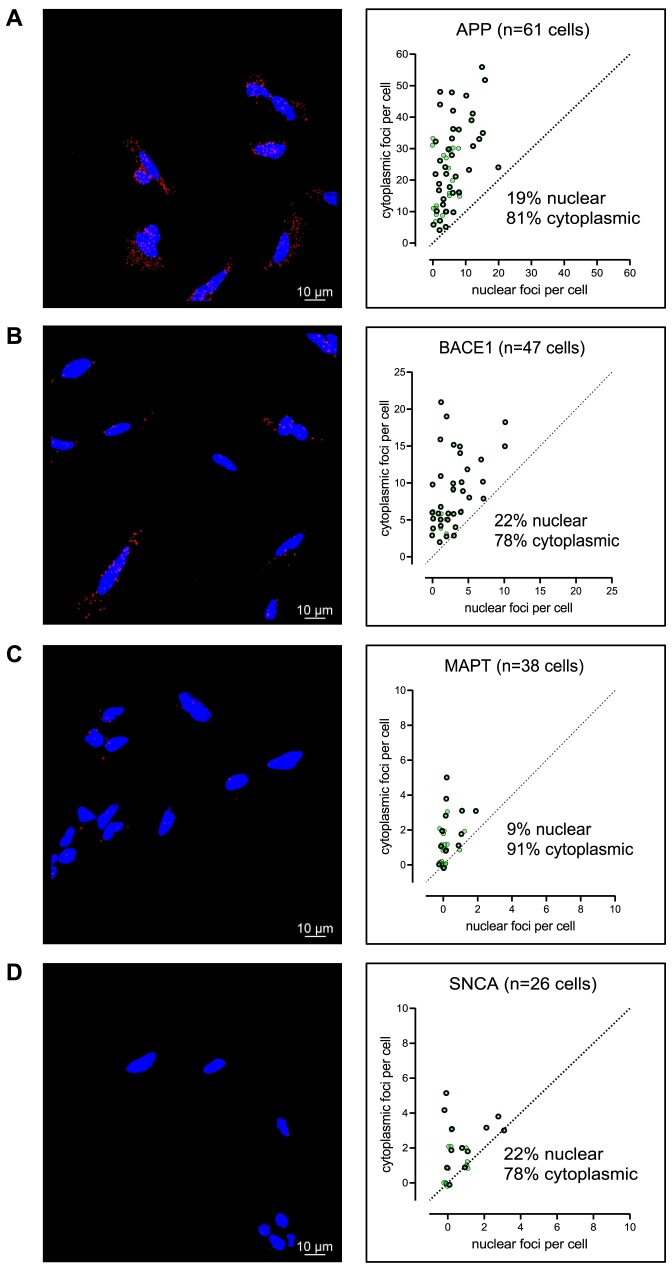
Intracellular localization does not contribute to siRNA efficacy in SH-SY5Y cells. RNAscope showing localization of (**A**) *APP*, (**B**) *BACE1*, (**C**) *MAPT*, or (**D**) *SNCA* mRNAs in SH-SY5Y cells. Left panels: representative images with target mRNAs (red) and nuclei stained with DAPI (blue); scale bar: 10 μm. Right panels: quantification of nuclear and cytoplasmic mRNA foci in SH-SY5Y cells (% of total foci).

The large discrepancies in the percentage of effective sequences (i.e. target expression ≤ 35%) across the four different targets in a native context ranged from 2% to 30% when using the more RISC-compatible 2′-OMe/-F scaffolds (Fig. 9, original sequences, native context, not filtered). In general, *BACE1* is the hardest to target. We have previously found that intracellular mRNA localization might impact the ability of RISC to find and cleave its mRNA target [49, 50]. In the case of *HTT* [50] and *ApoE* [49] mRNAs, nuclear-localized fractions were inaccessible to siRNAs, which resulted in overall lower target silencing. To evaluate if the level of target expression and intracellular localization impacts efficacy of siRNAs in our dataset, we used RNAscope [51] (i.e. advanced FISH method, see “Materials and methods” section) to examine the number and localization of individual mRNA foci within cells (Fig. 10). Although we did observe significant differences in expression levels—the average number of foci per cell is 28 for *APP*, 13 for *BACE1*, 2 for *MAPT*, and 3 for *SNCA*—these values do not correlate with hit rates for these genes, suggesting expression level alone is not predictive of hit rate.

Only a small fraction of each target mRNA (≤22%) was nuclear localized. Therefore, the differences in percentages of effective sequences across targets are not likely due to their nuclear localization in SH-SY5Y cells. However, given our previous observations for other neuronal targets [49, 50], nuclear localization is still an important factor to consider during siRNA design.

### Exon usage impacts siRNA efficacy

Alternative splicing is a common mechanism for gene regulation [52, 53], particularly for therapeutically relevant targets involved in neurodegeneration [54, 55]. Unfortunately, the top isoform reported by public databases (e.g. NCBI) does not always reflect the predominantly expressed isoform in disease-relevant models. Indeed, there are many isoforms reported for all four targets in this study (17 for *APP*, 12 for *BACE1*, 18 for *MAPT*, and 13 for *SNCA*, according to GTEx).

**Figure 11.**
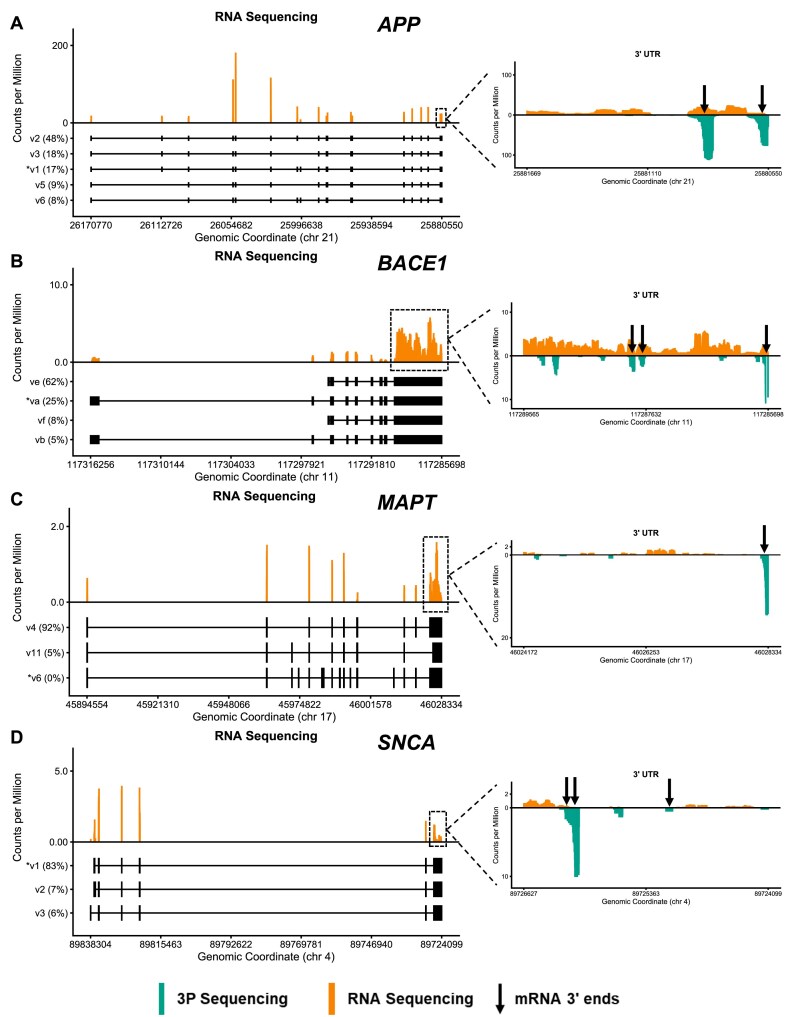
Exon usage and upstream PAS usage impact siRNA efficacy. RNA-seq data (left, right) and 3P-seq data (right) from the (**A**) *APP*, (**B**) *BACE1*, (**C**) *MAPT*, or (**D**) *SNCA* locus from undifferentiated SH-SY5Y cell RNA. Exonic read coverage from RNA-seq is shown in orange and read coverage from 3P-seq is shown in turquoise. All read counts are normalized by the total number of mapped reads in the libraries. On the left, gene bodies are shown for isoforms to which ≥5% of reads align according to RNA-seq data, and for the isoform used for siRNA design (marked with an asterisk). On the right, the 3′ UTR is shown for each gene. Black arrows mark the ends of qualified poly(A) sites (see “Materials and methods” section).

To evaluate if alternative exon usage might contribute to differential hit rates, we evaluated exon usage for SH-SY5Y cells by RNA sequencing. The results (Fig. 11, left panel, Supplementary Table S5) reveal that the predominantly expressed isoforms in SH-SY5Y cells differ from the main isoform used for siRNA design (marked with an asterisk) for *APP*, *BACE1*, and *MAPT*. A handful of siRNAs (five for *APP*, two for *BACE1*, and eight for *MAPT*) targeted underrepresented exons, explaining their lack of efficacy. However, this does not fully explain our siRNA silencing efficacy data (Figs 5–8). For example, the predominantly expressed *SNCA* isoform in SH-SY5Y cells, *SNCA* transcript variant 1, was used to design siRNAs; yet, there is a whole swath of siRNAs targeting the end of the 3′ UTR that were ineffective in a native context. Additionally, only a small number of the differences observed in reporter and native *BACE1* silencing are explained by exon usage; most of the differences are in the 3′ UTR. Thus, care should be taken to explore 3′ UTR usage, with preference given to sequences targeting transcript variants that utilize the most proximal polyadenylation site.

### The use of upstream poly-adenylation sites limits silencing by siRNAs targeting the ends of 3′ UTRs for some targets

Efficacy discrepancies between assays for siRNAs targeting the 3′ UTRs of target mRNAs (Figs 5–8) suggest there might be alternative PAS usage that cannot be derived from RNA-seq data alone. Indeed, alternative polyadenylation is heavily utilized in the brain, resulting in mRNA variants with 3′ UTRs of different lengths, and might be affected by cellular state [56–58]. To evaluate PAS usage in SH-SY5Y cells, we performed poly(A)-position profiling by sequencing (3P-Seq) (see “Materials and methods” section).

For *SNCA*, the large majority of reads map to upstream poly(A) sites, and for *BACE1*, ∼35% map to upstream poly(A) sites. siRNAs targeting these underrepresented regions would be unable to silence predominantly expressed isoforms in SH-SY5Y cells despite demonstrating RISC competence in a reporter context, explaining the discrepancy between assays. Interestingly, for *MAPT*, the downstream PAS is mainly used (Fig. 11), indicating that other factors—not PAS usage—account for limited silencing by siRNAs targeting the 3′ UTR of this gene.

After filtering out sequences that target underrepresented regions in the ORF and 3′ UTR (based on RNA-seq and 3P-seq data), hit rates generally improved for all targets, and most dramatically for *SNCA* (Fig. 9, Original Sequences, Native Context, Filtered versus not Filtered). These data indicate that experimentally validating the predominantly expressed isoform in the model system used for screening is critical.

**Figure 12.**
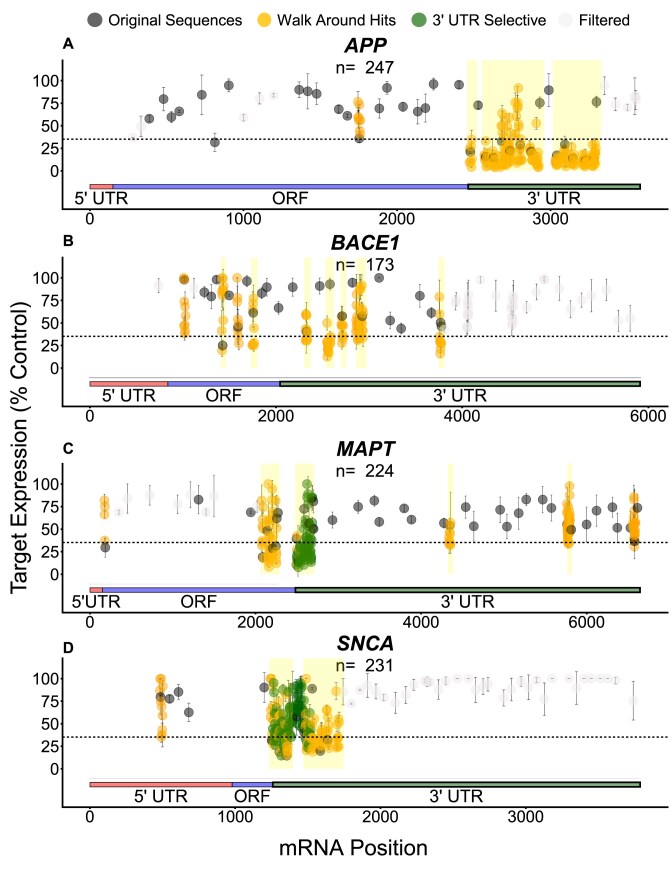
Effective sequences cluster together, and siRNA hit rates in the 3′ UTR are target dependent. siRNA target silencing results (*n* = 3, mean ± SD) in native context in Asymmetric 2′-OMe/-F scaffold for (**A**) *APP*, (**B**) *BACE1*, (**C**) *MAPT*, or (**D**) *SNCA*. SH-SY5Y cells treated for 72 h. Target mRNA expression levels measured using the QuantiGene 2.0 RNA Assay and calculated as percentage of untreated control. Datapoints are colored by dataset “Original Sequences” (dark gray), “Walk Around Hits” (orange), and “3′ UTR Selective” (green). Datapoints for siRNAs that don’t target prominently expressed mRNA regions according to RNA- and 3P-seq data are colored in off-white. Regions with high siRNA activity (i.e. “hot spots,” see “Materials and methods” section) are shaded in yellow. Dotted line at target mRNA expression = 35% to marks cutoff for siRNA hits. Gene regions (labelled) are shown below graphs.

To investigate potential differences in hit rate in the 3′ UTRs of genes without and with upstream PAS usage, we designed a panel of additional compounds (see “Materials and methods” section) selectively targeting the 3′ UTR of *MAPT* (no upstream PAS usage) or *SNCA* (upstream PAS usage) (Fig. 12, green dots). This strategy produced approximately three times as many hits for *MAPT* (90%) than for *SNCA* (34%), confirming certain regions in mRNAs can produce high hit rates.

### Effective target sequences cluster together

Given the significant contribution of local mRNA context on siRNA efficacy [14], we systematically explored the relative value of another conventional siRNA design strategy wherein a “walk” was performed around effective siRNA target sites (≤40% target mRNA expression in at least one of the scaffolds) identified in our original native screen. The “walk” panel of new siRNAs spanned 40-nt regions around the original effective target sites (see “Materials and methods” section). The sequences in this panel were exclusively synthesized in the Asymmetric 2′-OMe/ -F scaffold, as this pattern better accommodates RISC loading than the 2′-OMe rich pattern, and duplex structure had no impact on hit rate.

The results of the extended walks (Fig. 12, orange dots) show clear “hot spot” regions (*i.e*. clusters of effective siRNAs within 50 nts of one another, shaded yellow, see “Materials and methods” section) where most walks were performed. For all mRNA targets, the percentage of effective sequences increased compared to the original screen (*APP*: 36%–86%; *BACE1*: 4%–34%, *MAPT*: 10%–25%; *SNCA*: 38%–42%; see Fig. 9). For three of the four targets, this increase was statistically significant (*P*-values, Fisher’s exact test: *APP*= 0.001249, *BACE1*= 1.551e-07, *MAPT*= 0.01752, *SNCA*= 0.3339). Thus, these data demonstrate that conducting an additional walk around previously identified functional target sites is an effective strategy for identifying more functional siRNAs. Since the off-targeting profile of siRNAs is mostly determined by their seed sequence, closely located sequences can exhibit significantly different off-targeting signatures and potency.

### Translation efficiency correlates with siRNA silencing results

**Figure 13.**
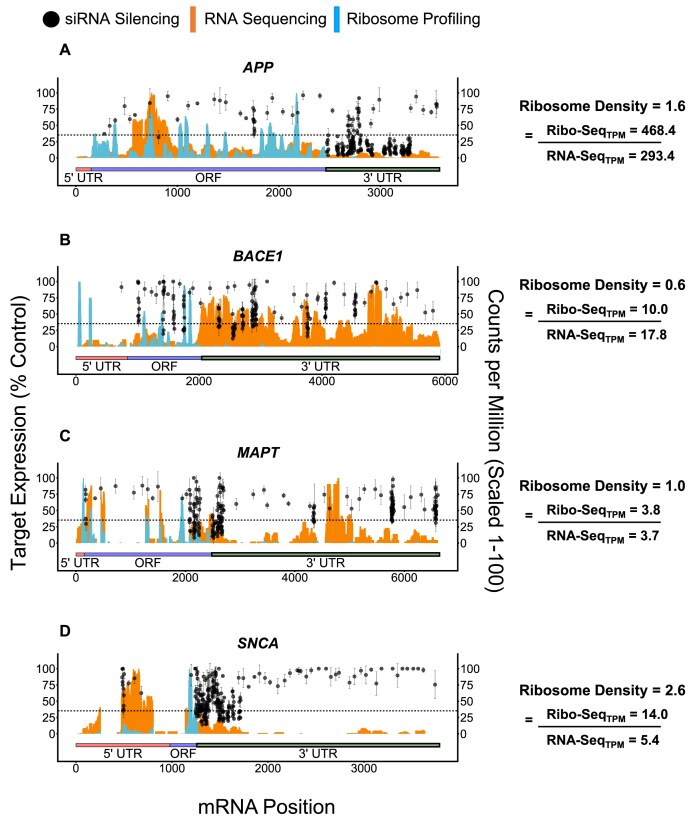
*APP* and *SNCA* are more efficiently translated than *BACE1* and *MAPT*. *MAPT* has a higher hit rate at the beginning of the 3′ UTR than *SNCA*. siRNA target silencing results (*n* = 3, mean ± SD) in native context in Asymmetric 2′-Ome/-F scaffold (black points), RNA-seq (orange), or ribosome profiling (blue) exonic read coverage for (**A**) *APP*, (**B**) *BACE1*, (**C**) *MAPT*, or (**D**) *SNCA*. For siRNA silencing, SH-SY5Y cells treated for 72 h, and target mRNA expression levels measured using the QuantiGene 2.0 RNA Assay and calculated as percentage of untreated control. Dotted line at target mRNA expression = 35% to marks cutoff for siRNA hits. For sequencing data, all read counts are normalized by the total number of mapped reads in the libraries. Exons (black and white) and gene regions (labeled) are shown below graphs. Ribosome density values are shown on the right.

We observed that, even after all corrections, the hit rate varies significantly between different target mRNAs, indicating they are primarily driven by mRNA-specific features. It is possible that ribosomes enable RISC function [59] by reducing local mRNA structural constraints to facilitate RISC binding. We therefore explored the potential relationship between target mRNA translation and siRNA efficacy, using ribosome density profiles from undifferentiated SH-SY5Y cells as a proxy for translation efficiency. Supplementary Figure S6 shows the resulting ribosome occupancy profiles for the four targets. Figure 13 plots siRNA efficacy (black datapoints) with a dotted line marking the cutoff for siRNA hits (i.e. ≤ 35% target mRNA expression) alongside read coverage for total RNA (orange) and ribosome occupied RNA (blue), and ribosome density (i.e. translation efficiency) calculated from these data on the right. Translation efficiencies range from 0.6 to 2.6, with *SNCA* and *APP* having the highest values and *BACE1* and *MAPT* having the lowest.

When considering only those siRNA sequences from our original dataset that target expressed isoforms, there is a statistically significant, positive correlation (*R*^2^= 0.91; *P* < 0.05, see Supplementary Fig. S7) between hit rate (*SNCA*: 38%; *APP*: 36%; *MAPT*: 10%; *BACE1*: 4%, Fig. 9) and translation efficiency (*SNCA*: 2.6; *APP*: 1.6; *MAPT*: 1.0; *BACE1*: 0.6, Fig. 13). For this limited number of targets, data indicate that highly translated mRNA have higher hit rates, which may be explained by different factors. Highly translated mRNAs may be more accessible to siRNAs, as sites of active translation could co-localize with the RISC complex. Other factors, such as mRNA location or interactions with other proteins, may positively influence both translation and siRNA activity. More detailed studies involving a larger number of targets are needed to determine if there is a general correlation between mRNA translation and success in siRNA-based modulation. Since translation efficiency can be significantly affected by the disease state, this factor may need to be considered when troubleshooting experiments where screening many siRNAs fails to yield an active hit.

### The use of reporter assay data builds a better predictive model of fully chemically modified siRNA efficacy than native context data

**Figure 14.**
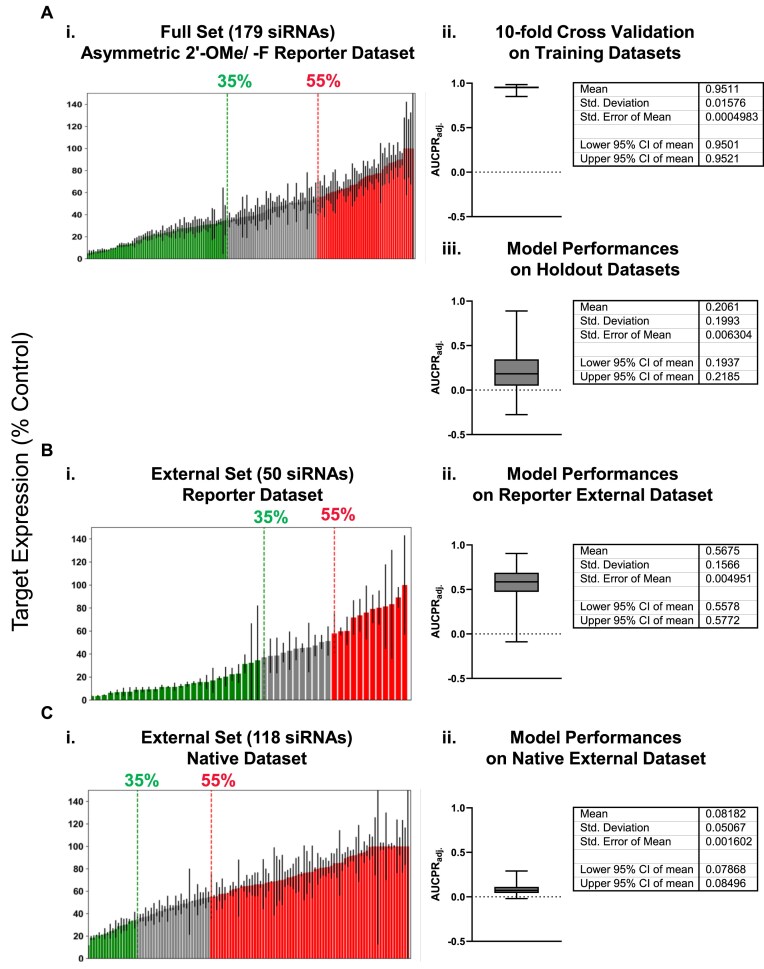
Machine Learning models trained on reporter assay data predict RISC-competence for fully chemically modified siRNAs. (**A–** **C**, left panels) siRNA (Asymmetric 2′-OMe/-F scaffold) target silencing results (*n* = 3, mean ± SD) for (A) the full dataset in a reporter context used to create the models and for (B and C) external datasets used to evaluate the models from (B) reporter or (C) native assays. Cells treated for 72 h. Target expression levels measured using the QuantiGene 2.0 RNA Assay (native) or Dual-Glo^®^ Luciferase Assay System (reporter) and calculated as percentage of untreated control. Dotted lines mark thresholds used for effective and ineffective siRNAs. (A–C, right panels) AUCPR_adj._ values plotted and statistics shown for each of the models generated from the training datasets (85% of the full dataset) for (Aii) 10-fold cross validations on the training datasets, and (Aiii) final model performances on the holdout datasets (15% of the full dataset), (Bii) reporter assay derived external dataset, and (Cii) native assay derived external dataset.

Because efficacy data from reporter assays explicitly describes the ability of RISC to silence its target independent of native context factors, it might be well suited to train algorithms predicting the RISC competence of siRNA sequences. To test this hypothesis, we built predictive models trained on the reporter assay data for sequences tested in the Asymmetric 2′-OMe/-F pattern (Fig. 14). These sequences are not biased by targeting location, this pattern better accommodates RISC loading, and asymmetric and blunt siRNA structures perform similarly. To build the models, we used a trichotomous partitioning method and random forest (RF) Machine Learning (ML) as described in Monopoli *et al.* [30] (see “Materials and methods” section), using single bases at each position in the target sequence as a feature. We chose these methods because they have previously demonstrated suitability for small datasets [30]. For each sequence, 50 nts (i.e. the 20-nt target site and 15-nt regions on each side of it) were extracted to train the models (see “Materials and methods” section). We chose to include the sequences flanking the target site because high adenine/uracil (A/U) content in these regions is an important feature of effective siRNAs [14], likely because it opens mRNA secondary structure and makes it available for interactions with RISC. Notably, the siRNAs evaluated here might generally be performing better in the context of the reporter assay because the plasmid inserts include only the 20mer target sequences strung together, which contain <40% Gs and Cs.

The analysis used two cutoffs to classify the 50-nt sequences into effective or ineffective groups reflecting biologically acceptable thresholds for effective and ineffective siRNAs (i.e. those resulting in ≤35% and >55% target expression, respectively) (Fig. 14Ai). These classified data were separated into model training and holdout sets, with equal distributions of effective and ineffective siRNAs in each set. This segmentation of the data was repeated 1000 times. Each holdout set consists of 15% of the dataset, is excluded from model training, and is used to evaluate model performance, which is assessed by a precision–recall (PR) curve. Precision represents the percentage of siRNAs correctly predicted to be effective with respect to all siRNAs predicted to be effective. Recall depicts the percentage of siRNAs correctly predicted as effective with respect to all effective siRNAs in the dataset. High precision (i.e. low number of false positives) is prioritized over high recall (i.e. low number of false negatives) because the goal of the predictive algorithm is to correctly identify effective siRNAs, not necessarily all possible effective siRNAs. Nevertheless, both parameters are important, which is why a higher area under the PR curve (AUCPR) generally indicates better model performance. However, this metric can be artificially inflated for models trained on different datasets because, at a certain threshold, a dataset with a higher proportion of effective sequences will have a higher AUCPR. To account for this, model performances were assessed using an adjusted metric called AUCPR_adj_, which is defined by subtracting the area defined by precision at maximum recall [30].

To determine whether the datasets and cutoffs are appropriate for model building, *K*-fold cross-validations (right panel, *K* = 10) were performed on each of the 1000 training datasets, wherein the data were randomly split into ten different groups and a model was trained on nine of those groups and evaluated on one of them iteratively. This provides a means to identify whether there are biases in the training data introduced by features in small numbers of sequences that might negatively impact algorithm performance before building the final models. Figure 14Aii plots results for 10-fold cross-validation of each training dataset, which are high and relatively consistent across all 1000 models, supporting the use of the selected cutoffs for final model building.

After using the training sets to generate models, models were evaluated using the holdout dataset (Fig. 14Aiii), an external dataset derived from reporter assay data containing fifty siRNA sequences with 14 different targets (Fig. 14Bi), and an external dataset derived from native (QuantiGene) assay data with more than one hundred siRNA sequences targeting six different genes in four cell lines (Fig. 14Ci). Our algorithms showed reasonable predictive power for the holdout (average AUCPR_adj_ = 0.2061, Fig. 14Aiii) and external reporter (average AUCPR_adj_ = 0.5675, Fig. 14Bii) datasets, but performed worse when tested on the external native dataset (average AUCPR_adj_ = 0.08182, Fig. 14Cii).

To better predict efficacy in a native context, we built models using our native dataset. While the hit rate for this dataset was low (Fig. 9, Original Sequences, Native Context, Asymmetric 2′-OMe/-F), the numbers were reasonable enough to perform a similar analysis. We built models using both the full dataset (Supplementary Fig. S8Ai) and the filtered dataset that includes only siRNAs that target prominently expressed isoforms according to RNA- and 3P-seq data (see “Materials and methods” section) (Supplementary Fig. S9Ai). Results for 10-fold cross-validations were acceptable for both datasets (Supplementary Figs S8Aii and S9Aii). The models built on the full dataset performed worse than the models built on the filtered dataset when evaluated on the holdout sets (average AUCPR_adj_= 0.1421 versus average AUCPR_adj_= 0.2586, Supplemental Figs S8Aiii and S9Aiii, right panels). Based on our earlier analyses, we know that the full dataset contains improperly classified sequences, i.e. sequences that are effective but appear ineffective because they target under-represented isoforms in SH-SY5Y cells. Removing such sequences improves the model, suggesting they impede the construction of a model that can define effective siRNAs targeting the four genes tested. We tested each model type on an external dataset derived from native assay data and observed that both performed poorly (average AUCPR_adj_ = 0.01868 for the full native dataset and average AUCPR_adj_ = 0.03935 for the filtered native dataset, Supplemental Figs S8B and S9B).

Notably, the hits in the filtered, native dataset constitute 20% of the total number of sequences, while the hits in the reporter dataset constitute 42%. To clarify whether the lower performance of the predictive model trained on the filtered, native dataset was due to the smaller proportion of true hits, we randomly under-sampled the true hits in the reporter dataset to match the proportion of true hits in the native, filtered dataset and built new models (Supplementary Fig. S10). Similar to the models trained on the full reporter dataset, these models performed relatively well on the external reporter dataset (average AUCPR_adj_ = 0.3789, Supplementary Fig. S10B) and worse when tested on the external native dataset (average AUCPR_adj_ = 0.04339, Supplementary Fig. S10C). This suggests that model performances were not dependent on the proportions of hits in the training data.

Collectively, these data suggest reporter-derived data are well suited for building models to predict RISC-competence of fully chemically modified siRNAs, but not to predict efficacy in a native environment because there are factors impacting efficacy that cannot be accounted for using only the target sequence as a feature.

**Figure 15.**
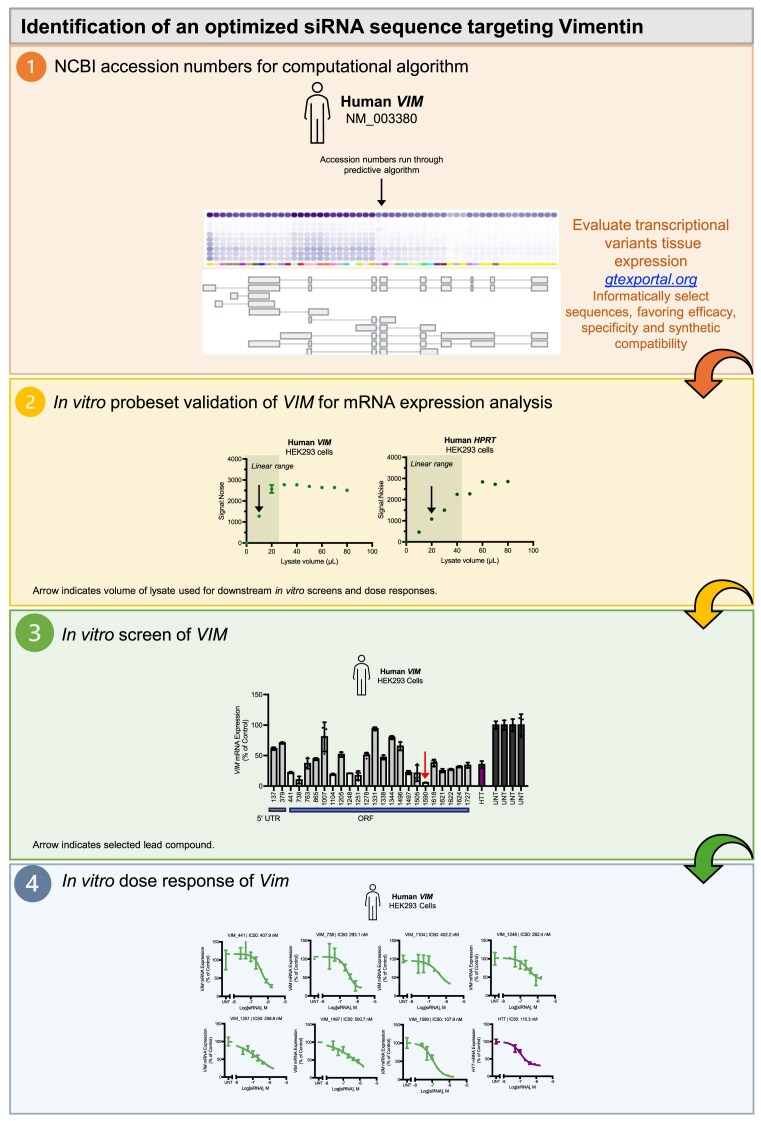
Flow chart for the design and identification of siRNA targeting vimentin. (1) Accession number is identified. The GTEx portal evaluates transcriptional variants and expression in tissues of interest. siRNAs are designed to favor factors known to affect RISC entry, specificity, and synthetic compatibility. (2) Cell lines expressing the target of interest are identified, and assays are validated to determine the linearity of the assay range (QuantiGene). (3) The primary screen is performed at high concentrations to identify lead compounds. (4) The efficacies of the lead compounds are validated in a dose–response study, and IC_50_ values are determined.

Finally, we present a model workflow for the successful design of chemically modified siRNAs (Fig. 15). Here we show the steps, from informatically selecting target sequences to analyzing the target transcript isoform usage in the context of a biologically relevant cell line and validating assays for the measurement of siRNA activity, to screening for hit compounds, and finally, to validating and choosing a lead compound using dose response experiments. This workflow provides a foundation from which to begin, but other steps, such as testing an siRNA “walk” around hit compounds and validating lead compounds in the tissue of interest *in vivo* can also be included in the screening and lead selection workflow.

## Discussion

Chemically modified siRNAs are a maturing modality for both the study of gene function *in vivo* as well as therapeutic intervention for genetically defined diseases. Still, they are a complex reagent for which a clear set of guidelines for effective design and validation is lacking. Here, we present key aspects to be considered to enable the identification of potent siRNAs in the context of four therapeutically relevant targets as well as examples of how to recognize and avoid design pitfalls. While biotechnology and pharmaceutical companies interested in developing siRNA therapeutics may have the resources to test many siRNA sequences and designs, the daunting nature of this task has limited the widespread use of chemically modified siRNAs for use in basic biology research. This work provides a framework which can be used to make these design efforts more efficient and fruitful, thus making the use of chemically modified siRNAs more approachable for researchers.

### Cell type matched transcript isoform information is essential for siRNA design

We show profound differences in hit rates for each target when screening siRNAs in a native context (Fig. 9, Original Sequences, not Filtered), demonstrating the need to better understand the biology of the target mRNA before designing and screening siRNA. For example, we show that exon and PAS usage influences the overall silencing efficacy of siRNAs targeting these regions of the mRNA. Both PAS and exon usage might not be apparent on publicly available databases like NCBI and GTEx, but there is an abundance of publicly available RNA-seq datasets from popular cell culture models that can be analyzed to determine exon usage. The length of the 3′ UTR for prominent isoforms is also crucial to consider because many cell culture models used for siRNA screens are derived from cancer patients, and 3′ UTR shortening is characteristic of cancer cells [56]. When alternative PAS usage is not evident from RNA-seq data alone, as was the case in the present study, it may be advantageous to assume the most upstream PAS is being used to increase the probability of identifying effective sequences.

### Reporter assays may not accurately represent siRNA efficacy in the native mRNA context

For some target mRNAs, we observe significant discrepancies between efficacy in reporter assays and native contexts, even after accounting for alternative isoform expression. Reporter assays are commonly used as a proxy to identify functional siRNAs, particularly for therapeutic targets with limited expression in widely used cell types or those requiring specific treatments for activation. While it is acceptable to use reporter assays for primary screening, the efficacy of identified hits must be validated in a biologically relevant context. Reporter assays only confirm that a compound can enter the RISC complex and silence the target in an idealized, isolated environment, they do not guarantee efficacy in the native context or account for mRNA-specific features.

### siRNA sequence and chemical modifications must be considered in tandem

While siRNA duplex structure may contribute to pharmacokinetic/pharmacodynamic (PK/PD) profiles [16], our data show that structure does not impact hit rates or efficacy *in vitro* (Figs 1 and 2). Therefore, different structures can be used for siRNA screening, and the hits identified in one structure will likely translate to another.

Unlike siRNA structure, siRNA chemical pattern has a profound impact on hit rates and target silencing efficacy (Figs 1 and 3). Although 2′-OMe is more stable than 2′-F [42], the 2′-OMe Rich pattern selected for this study is tolerated by RISC in a smaller subset of sequences, as evidenced by much lower hit rates in the context of the reporter assay (Fig. 1). Most sequences that are effective when synthesized in the 2′-OMe Rich pattern will work in the 2′-OMe/-F optimized patterns but not vice versa (Fig. 3). Compared to 2′-OMe/-F restrictive sequences, 2′-OMe Rich permissive sequences have lower thermodynamic stability in the regions surrounding the siRNA target site (Fig. 3). This has been previously reported for effective targeting sequences [14], and it is possible that more effective sequences simply better counteract the negative impacts of 2′-OMe on RISC function. However, this sequence feature might also be related to RISC kinetics or other factors more specific to interactions between the sequences and 2′-OMe Rich scaffold. For example, more flexibility around the target site might allow RISC to better accommodate siRNAs in the 2′-OMe Rich pattern, which do not conform as closely to the structure of unmodified siRNA as 2′-OMe/-F patterned siRNA. 2′-OMe Rich patterns can have profoundly positive impacts on *in vivo* efficacy based on longer-termed stability [18], but finetuning the exact chemical pattern to different sequences is important. Indeed, when an effective target site is identified, the IC_50_ value is similar across the two patterns (Supplementary Fig. S4).

These data suggest there may be limits to developing the siRNA sequence and chemical scaffold separately. Although this approach may be possible for the most effective sequences, the relationship becomes more complicated for moderately effective sequences (see Fig. 3D). The reason for this is unknown, but one can speculate that it is dependent on individual or combined nucleotide interactions with RISC that are specific to each sequence. If moderately effective sequences are limited by RISC competence, they would be particularly sensitive to chemical scaffolds that further disrupt interactions with RISC. It is therefore important to understand one's individual needs when designing siRNAs. If relatively short-term efficacy is the most important parameter, using a slightly less stable but more RISC competent modification pattern will provide the best results. If long-term durability is of the utmost importance, however, designing a chemical modification pattern that maximizes metabolic stability while remaining active with the sequence of choice is necessary. Iterative design of chemical modification patterns, beginning with the most permissive and gradually transitioning to more stable, but potentially less permissive patterns, enables the optimization and identification of the best sequence-modification pattern combination. Indeed, different modification patterns are utilized in approved GalNac siRNAs.

### Efficacious siRNAs tend to cluster together along a target transcript

The extended “walk” screens performed around effective siRNAs increased hit rates (Fig. 9) and revealed “hot spots” (Fig. 12, yellow shaded regions) of effective siRNAs, in all four target mRNAs. This widely used strategy could, therefore, prove useful when primary screens yield low numbers of effective siRNAs. It is unlikely that every 20-nt siRNA target sequences in mRNA “hot spots” are ideal for RISC function, given that shifting the siRNA start site by a single base position in the mRNA sequence can significantly change the degree of silencing observed (e.g. SNCA_1347_Asymmetric_2′-OMe/-F results in 83% target expression, while SNCA_1348_Asymmetric_2′-OMe/-F results in 22% target expression, Supplementary Table S1). However, local mRNA structure may, in general, be favorable to RISC in these regions. Strong preferences for A or U in the ≤15 nucleotide regions surrounding the target site have been observed previously [14], but the “walk” screen results suggest that the secondary structure distal to the target site is important for RISC function, too. Alternatively, these mRNA hotspots may be in near other RNA-binding proteins or microRNA (miRNA) sites, which enhances the efficacy of all RISC-entering compounds in these regions.

### Target transcript expression level does not substantially influence siRNA efficacy

We saw no correlation between hit rate and level of target mRNA expression (Figs 9 and 10, Original Sequences, Native context), but target hit rate was significantly correlated to translation efficiency—genes with higher translation efficiencies have the highest hit rates (Figs 9 and 13, Original Sequences, Native context, Supplementary Fig. S7). These results align with published data showing translation enhances RISC activity [59]. Higher numbers of ribosomes might underlie the higher hit rates for more highly translated genes, but other factors may influence hit rates in different regions of the mRNA. For example, the degree to which individual mRNAs are structured may influence the proposed model [59], with highly structured regions reaping more benefits from the presence of ribosomes than less structured ones. Our observations are worth exploring on a larger scale and in more depth. To investigate the apparent correlation between ribosome density and siRNA silencing, one could induce changes in ribosome occupancy from low to high, or vice versa, and measure changes in siRNA-mediated silencing across the lengths, and within specific regions, of mRNAs with varying secondary structures.

### Algorithms trained on reporter assays have limited predictive power for silencing of endogenous transcripts

Monopoli *et al.* [30] recently reported an RF ML model that can be trained on relatively small datasets for chemically modified siRNA sequence selection. The current manuscript extends these findings by describing how to make use of that tool most effectively. We show that the RF ML model successfully predicted siRNA efficacy when trained on reporter assay data, indicating its usefulness in predicting RISC-competent sequences. However, the model performed poorly when trained on native context data, suggesting it is more challenging to algorithmically account for target mRNA biology factors impacting siRNA efficacy. Thus, it may be advantageous to use such a model to first identify RISC competent chemically modified siRNA sequences for a target mRNA. RISC-competent sequences can then be filtered based on target-specific factors in the relevant native context. Future studies utilizing larger datasets, advanced AI-based tools, and genomic context information may be needed to develop more accurate siRNA efficacy prediction models. These models would need to combine the ability to assess siRNA productive RISC interactions and to accurately identify “RNAi-sensitive” mRNA locations.

Although we identified multiple native context factors impacting siRNA-mediated mRNA silencing, there are undoubtedly other factors that may be important. For example, prior reports suggest mRNA with rapid turnover could be more difficult to silence than those with slower turnover rates [60], providing further evidence that maximum silencing efficacy is target specific and likely driven by mRNA features. Large data sets comprising siRNA efficacy (in native context) and mRNA turnover results for diverse compounds and targets should be analyzed in a comprehensive manner. As more target-related determinants of siRNA efficacy are defined, more complex siRNA efficacy algorithms that incorporate native context factors as model features could be developed to further simplify therapeutic siRNA design.

### Experimental example using design strategy

Based on our findings, we propose the following strategies to consider for design and execution of a siRNA screening project (Fig. 15):

- **Select target sequences**: Run siRNA predictive algorithms trained on reporter assay data, which evaluate positional base preferences to predict RISC competency. Alternatively, basic rules previously summarized could be used [22].
- **Define model system and exon and PAS usage:** Identify prominent target isoforms and define exon and PAS usage (if data are available) for therapeutically relevant cells and tissues. Filter the list of predicted sequences from step one based on these findings. If no PAS data are available, favor sequences targeting the shortest predicted 3′ UTR.
- **Perform *in vitro* screen:** Identify lead compounds by screening target sequences.
- **Perform follow up *in vitro* dose response:** Validate lead compound efficacy in a dose response study.
- **Optional:** If an insufficient number of active compounds is identified, consider designing a “walk-around” strategy for the active sites. This involves creating a panel of siRNAs that shift the position by 1–2 nucleotides to the right and left of the identified lead.

The following parameters can also be considered:

- **Choose siRNA scaffold**: Scaffold selection should be based on the therapeutic application (e.g. target tissue and duration of effect). Using RISC-competent patterns (e.g. 2′-OMe/-F) allows for the identification of a higher number of hits, while less RISC-competent patterns (e.g. 2′-OMe-rich) may identify the most effective sequences or those most likely to tolerate a broader range of chemical modification patterns. Depending on the goal, a more permissive scaffold increases the likelihood of finding functional compounds, whereas a more restrictive scaffold facilitates the identification of highly active sites that are likely to tolerate a wide range of chemical modifications.
- **Consider intracellular localization:** Consider the intracellular location of targets, as nuclear-localized targets may be more challenging to silence on the mRNA level. In such cases, evaluating siRNA efficacy by measuring target protein levels can serve as an indicator of cytoplasmic mRNA silencing. Since only cytoplasmic mRNAs contribute to protein translation, using protein levels as a readout can provide a higher signal-to-noise ratio.

These guidelines can assist researchers in identifying effective siRNAs, potentially speeding up the development of siRNA drugs and troubleshooting cases where screens failed to identify effective compounds.

## Supplementary Material

gkaf479_Supplemental_Files

## Data Availability

Sequence Data Resources 3P-seq data available on GEO under accession number GSE231101 (https://www.ncbi.nlm.nih.gov/geo/query/acc.cgi?acc=GSE231101).
