# Supplementary material for: Systematic analysis of siRNA and mRNA features impacting fully chemically modified siRNA efficacy
Source: Nucleic Acids Res. 2025 Jun 23;53(12):gkaf479. doi: 10.1093/nar/gkaf479 (PMC12205987; doi:10.1093/nar/gkaf479)
Supplement: gkaf479_Supplemental_Files [file gkaf479_supplemental_files.zip › Supplemental Figures.docx]

**
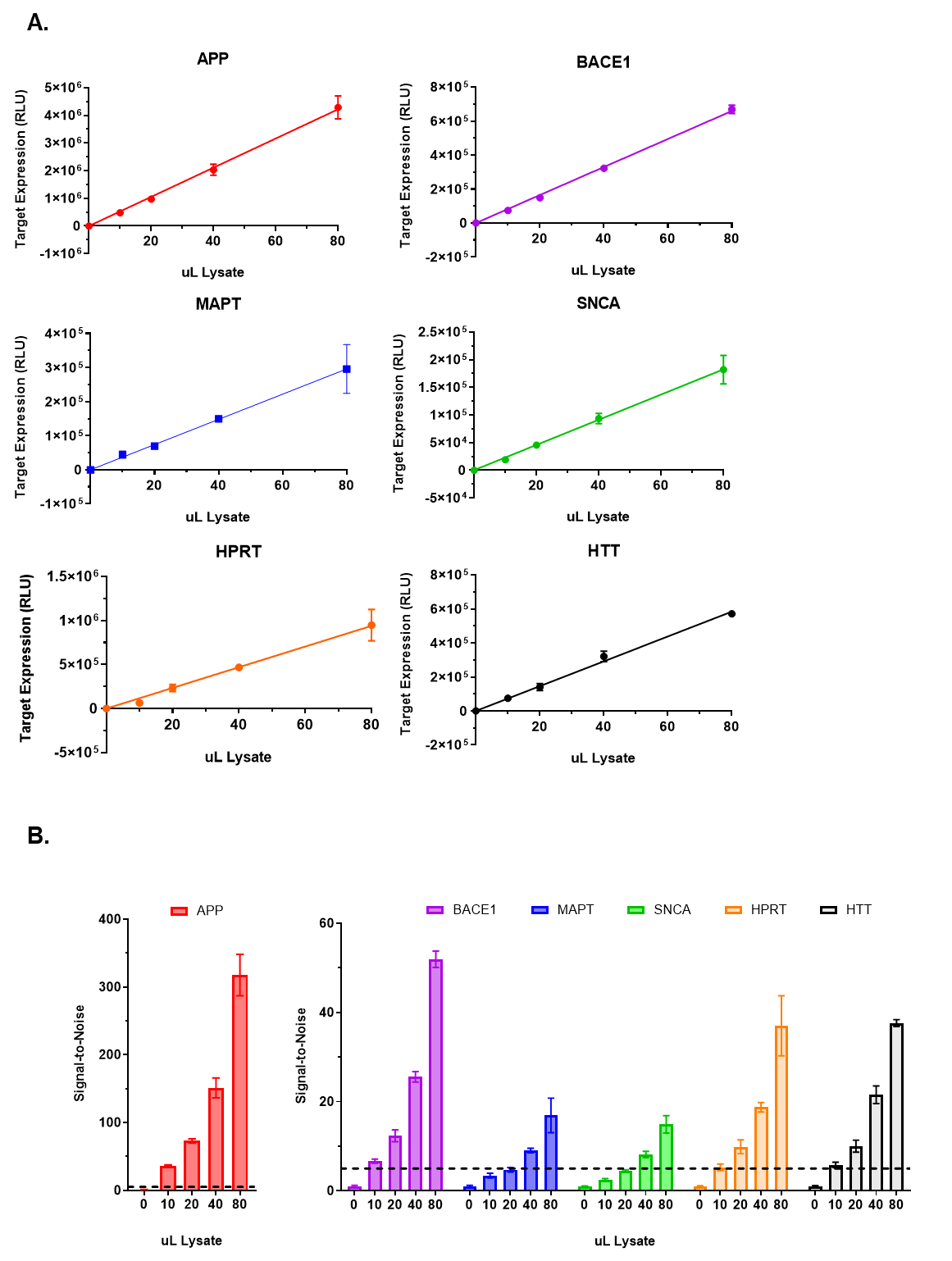
**

**Supplemental Figure 1**. **QuantiGene 2.0 RNA Assay probe sets demonstrate linear response and appropriate signal-to-noise**

Probe set validation results (n=3, mean ± SD). Target name indicated in each graph. SH-SY5Y cells plated and grown for 72 hours. Amount of QuantiGene 2.0 RNA Assay probe set was held constant while cell lysate amounts (indicated on x-axis) were increased. The resulting (A) target mRNA expression levels and (B) signal-to-noise ratios (dotted line marking the lower-limit cutoff = 5) were measured.


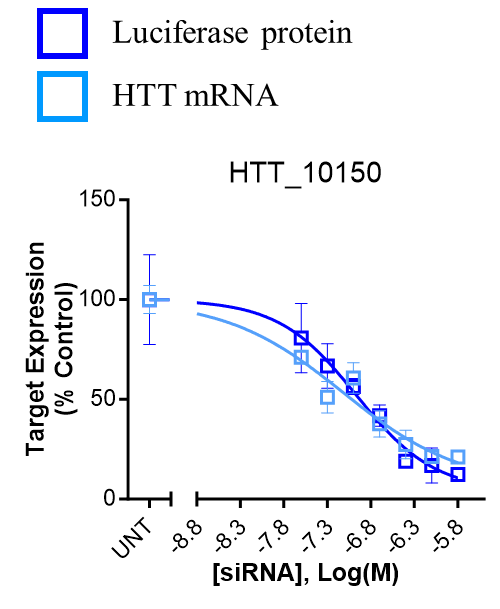


**Supplemental Figure 2. Luciferase mRNA and protein levels show similar results**

Concentration response results (n=3, mean ± SD). Average untreated (i.e., UNT) value (i.e., 100%) for entire test plate plotted. Target name and start site of target sequence indicated. HeLa cells treated with siRNAs at concentrations shown for 72 hours. Target expression levels measured using the Dual-Glo® Luciferase Assay System (protein) or QuantiGene 2.0 RNA Assay (mRNA) and calculated as percentage of UNT control. HTT compound information in **Supplemental Table 6**.

**
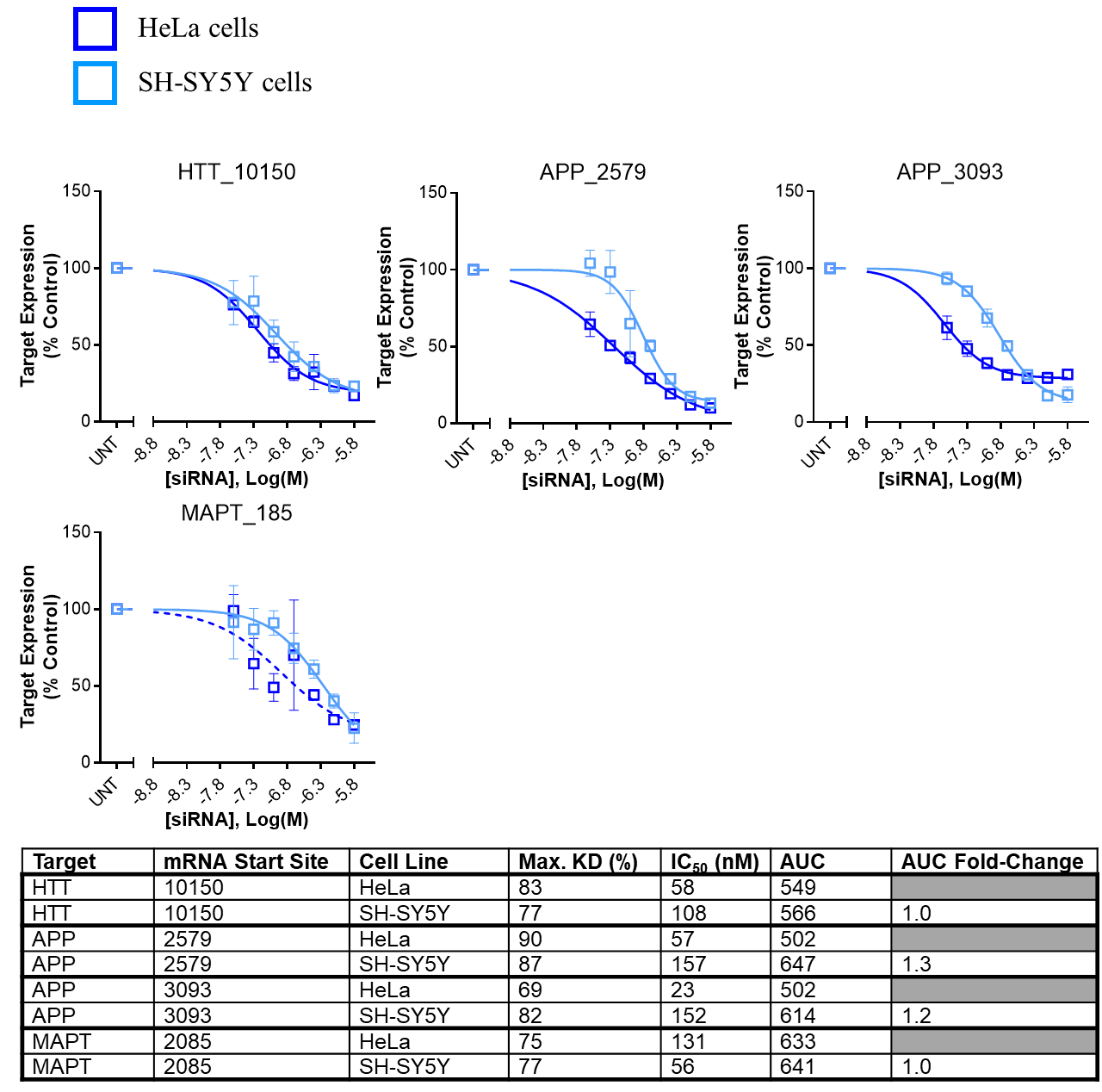
**

**Supplemental Figure 3. Concentration dependent response shows similar mRNA silencing in HeLa and SH-SY5Y cells**

Concentration response results (n=3, mean ± SD). Average untreated (*i.e.*, UNT) value (*i.e.*, 100%) for entire test plate plotted. Target name and start site of target sequence indicated in each graph. HeLa and SH-SY5Y cells treated with siRNAs at concentrations shown for 72 hours. Target expression levels measured using the QuantiGene 2.0 RNA Assay System and calculated as percentage of UNT control. Non-linear regression curves with R^2^<0.8 displayed as dashed lines. The table below reports values corresponding to graph: maximum average mRNA expression with top treatment dose of siRNA (% untreated control), IC_50_ - half maximal inhibitory concentration, and AUC - area under the dose response curve, and AUC fold-change between each set of results. *APP* and *MAPT* compound information in **Supplemental Table 1**, *HTT* compound information in **Supplemental Table 6**.

**
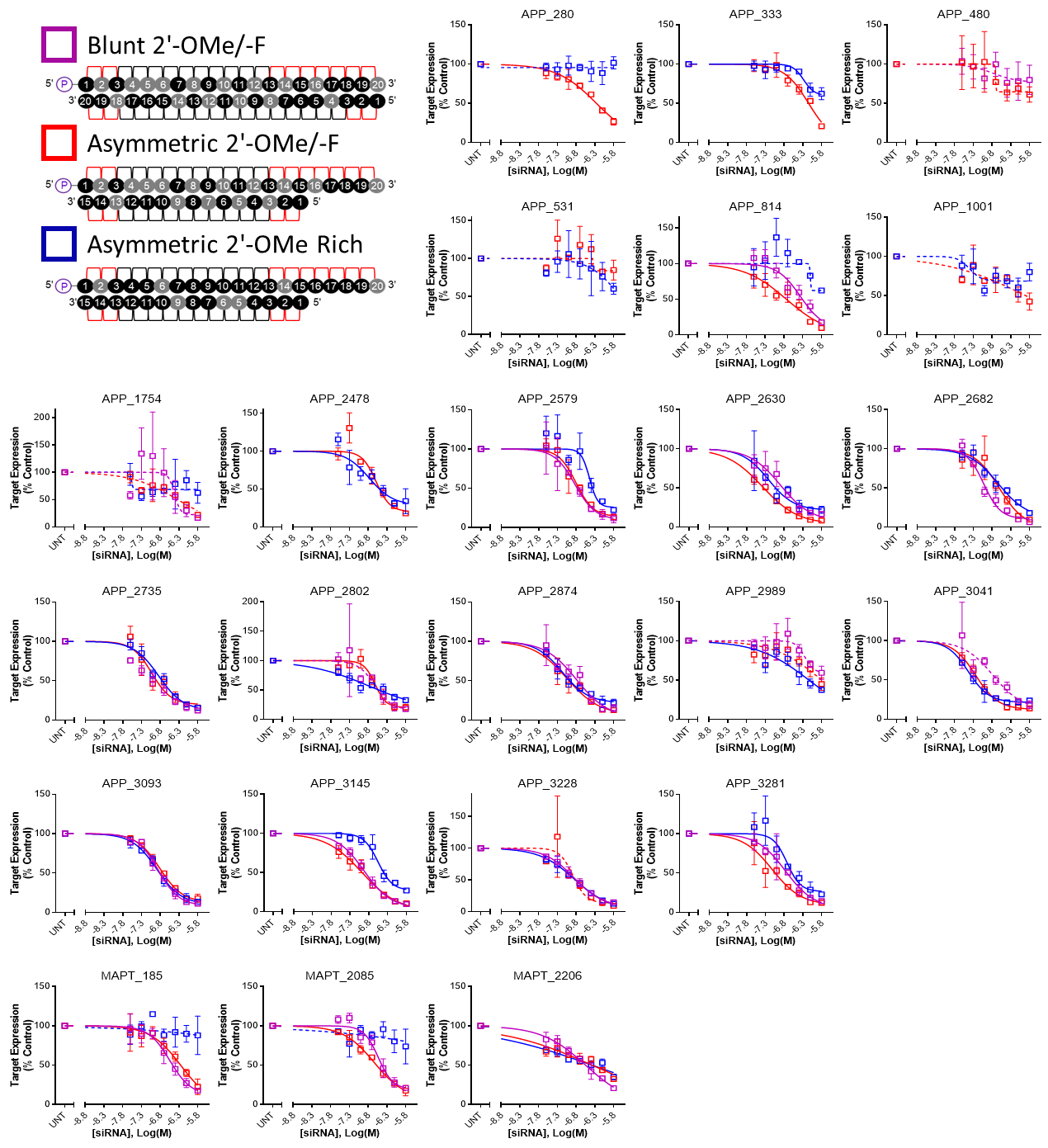
**

**Supplemental Figure 4. Concentration dependent response follows trends observed in single point efficacy experiments**

Concentration response results (n=3, mean ± SD). Average untreated (*i.e.*, UNT) value (*i.e.*, 100%) for entire test plate plotted. Target name and start site of target sequence indicated in each graph. SH-SY5Y cells treated with siRNAs at concentrations shown for 72 hours. Target mRNA expression levels measured using the QuantiGene 2.0 RNA Assay and calculated as percentage of untreated control. Non-linear regression curves with R^2^<0.8 displayed as dashed lines. **Supplemental Table 2** reports values corresponding to graph: maximum average mRNA expression with top treatment dose of siRNA (% untreated control), IC_50_ - half maximal inhibitory concentration and corresponding R^2^ value, and AUC - area under the dose response curve.


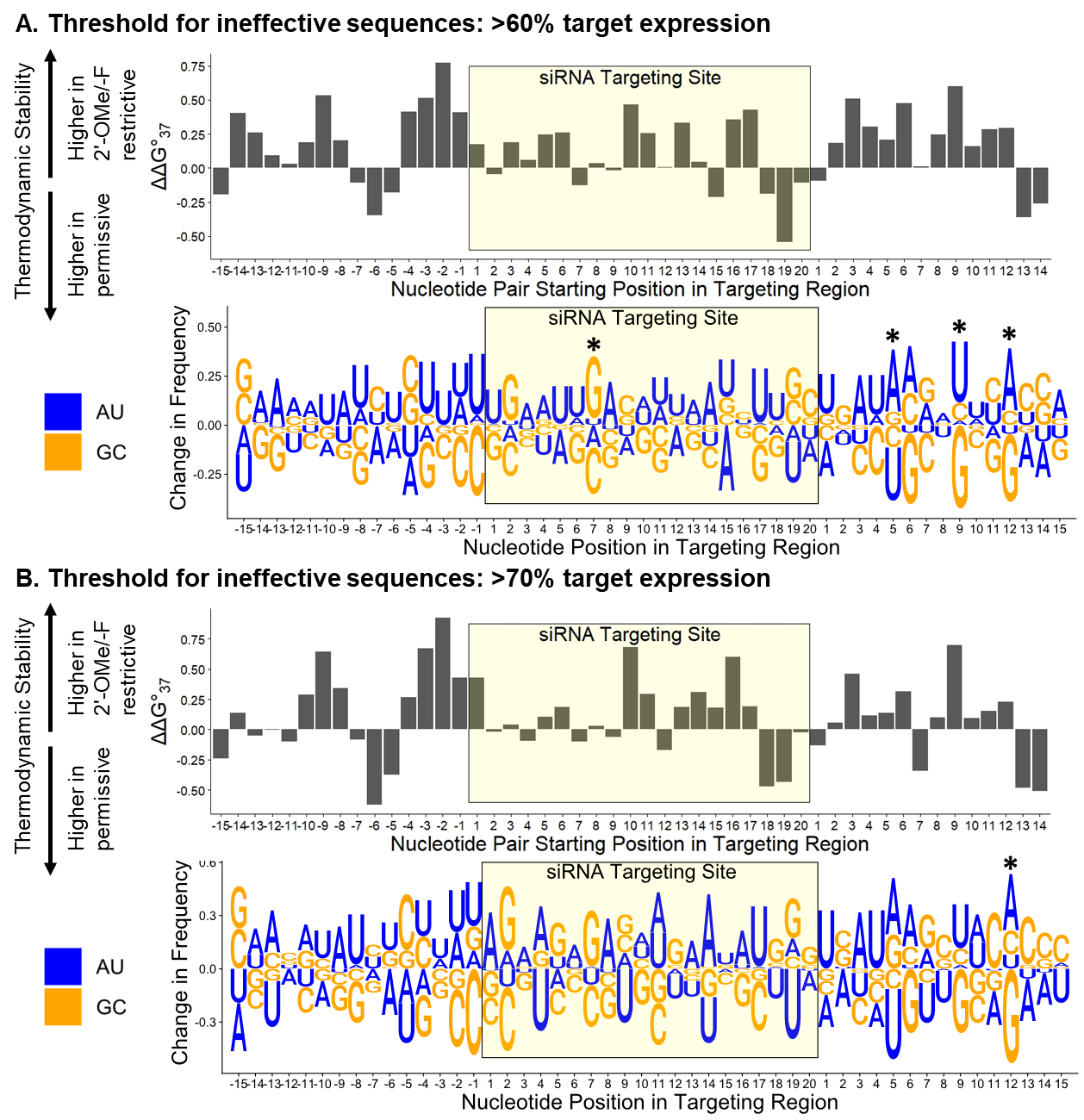


**Supplemental Figure 5. Increasing the threshold for ineffective sequences does not change the sequence trends observed for siRNAs with different chemical patterns**

Changes in thermodynamic stability (*i.e.*, ΔΔG ̊37, top panels) and nucleotide frequency (lower panels) between the pattern permissive (*i.e.*, sequences causing ≤35% target expression in both scaffolds) and Asymmetric 2′-OMe/-F pattern restrictive – *i.e.*, sequences causing ≤35% target expression in the Asymmetric 2′-OMe/-F pattern and (A) >60% or (B) >70% target expression in the Asymmetric 2′-OMe Rich pattern groups. HeLa cells treated for 72 hours. Target expression levels (n=3, mean ± SD) measured using the Dual-Glo® Luciferase Assay System and calculated as percentage of untreated control. ΔΔG ̊37 plotted for each nucleotide pair in the 50mer targeting region, with each position number marking the first position of each nucleotide pair. p-values describe statistically significant differences between groups (t-test with Benjamini-Hochberg correction; non-significant differences unmarked). Change in nucleotide frequency plotted for each nucleotide in the 50mer targeting region. p-values describe statistically significant differences between groups (Fisher’s exact test; * p<0.05, non-significant differences unmarked).


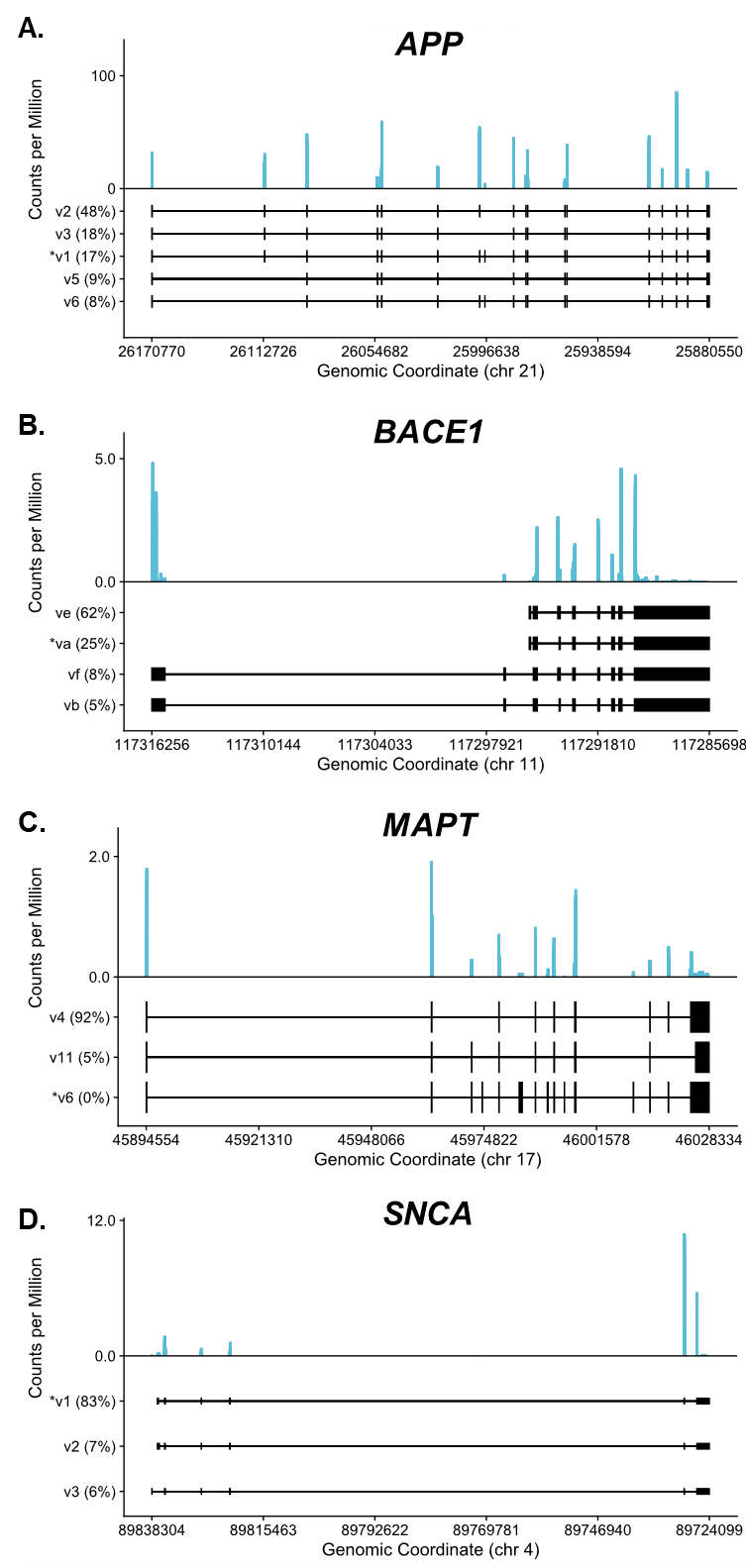


**Supplemental Figure 6. *APP* and *SNCA* have higher ribosome occupancy than *BACE1* and *MAPT***

(A-D) Ribosome profiling data from the (A) *APP*, (B) *BACE1*, (C) *MAPT*, or (D) *SNCA* locus from undifferentiated SH-SY5Y cell RNA. Exonic read coverage is shown in light blue. All read counts are normalized by the total number of mapped reads in the libraries. Gene bodies are shown for isoforms to which ≥5% of reads align according to RNA-seq data, and for the isoform used for siRNA design (marked with an asterisk).


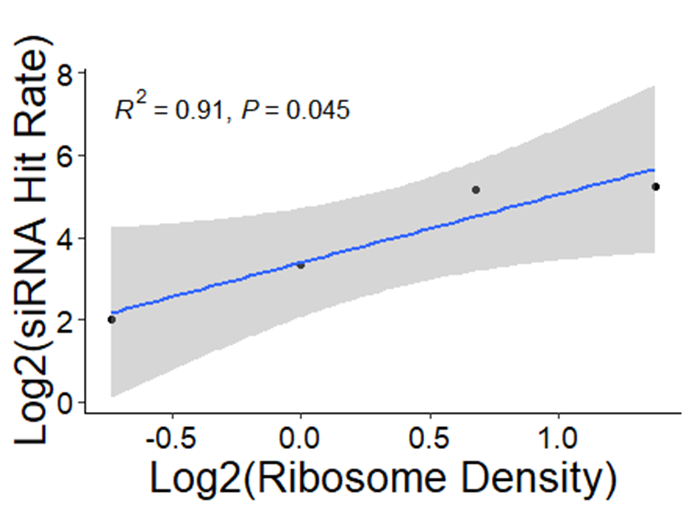


**Supplemental Figure 7. siRNA hit rate and ribosome density show a positive correlation**

Log_2_ values plotted for siRNA hit rate (y-axis) and ribosome density (x-axis) for all four target mRNAs. P-value was calculated using linear regression.


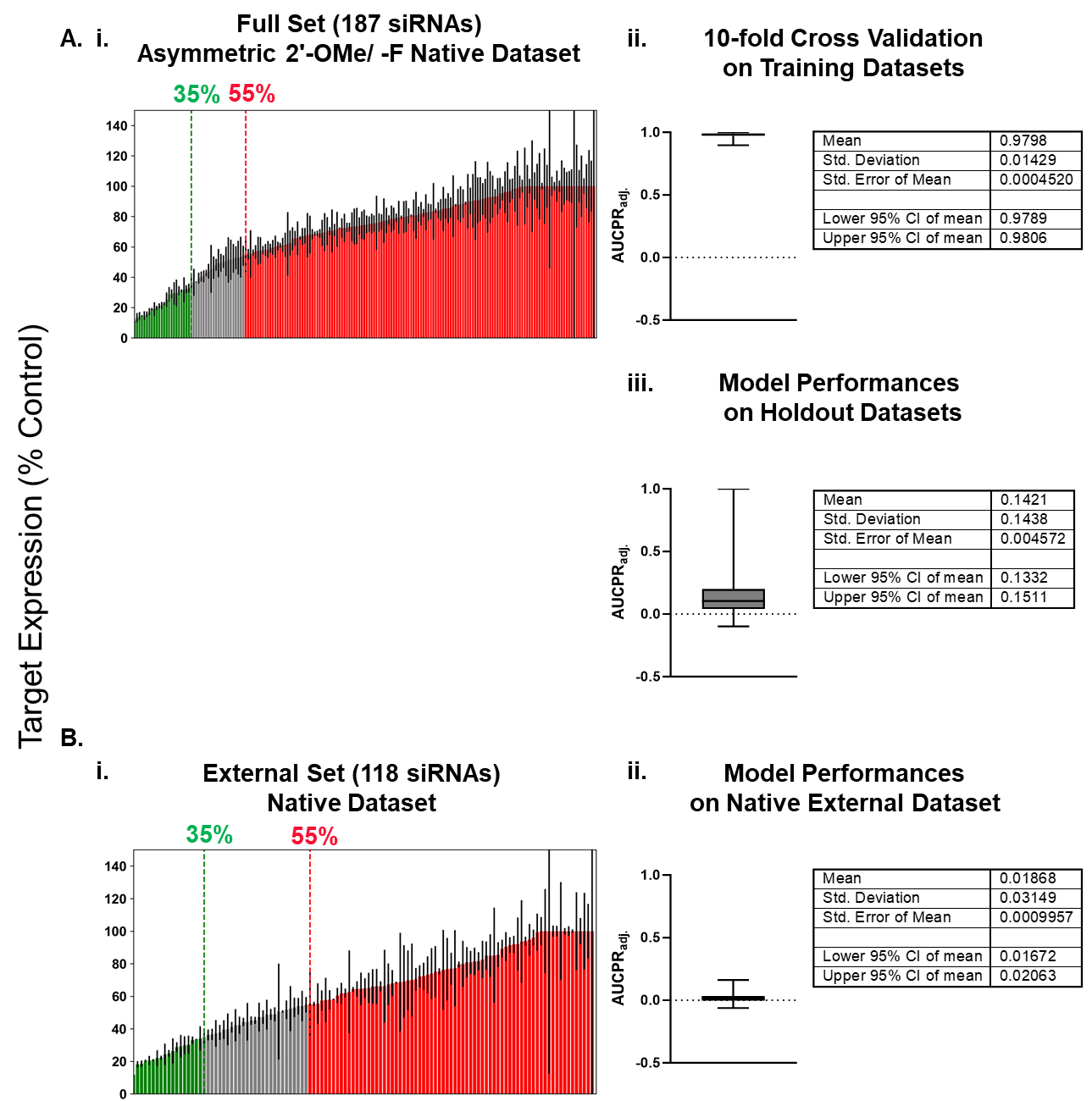


**Supplemental Figure 8. Machine Learning models trained on native assay data poorly predict fully chemically modified siRNA efficacy for other targets in external dataset**

(A and B, left panels) siRNA (Asymmetric 2′-OMe/-F scaffold) target silencing results (n=3, mean ± SD) for (A) the full dataset in a native context used to create the models and for (B) the external dataset excluded from model building derived from native assay. Cells treated for 72 hours. Target mRNA expression levels measured using the QuantiGene 2.0 RNA Assay and calculated as percentage of untreated control. Dotted lines mark thresholds used for effective and ineffective siRNAs. (A and B, right panels) AUCPR_adj._values plotted and statistics shown for each of the models generated from the training datasets (85% of the full dataset) for (Aii) 10-fold cross validations on the training datasets, and (Aiii) final model performances on the holdout datasets (15% of the full dataset) and (Bii) native assay derived external dataset.

**
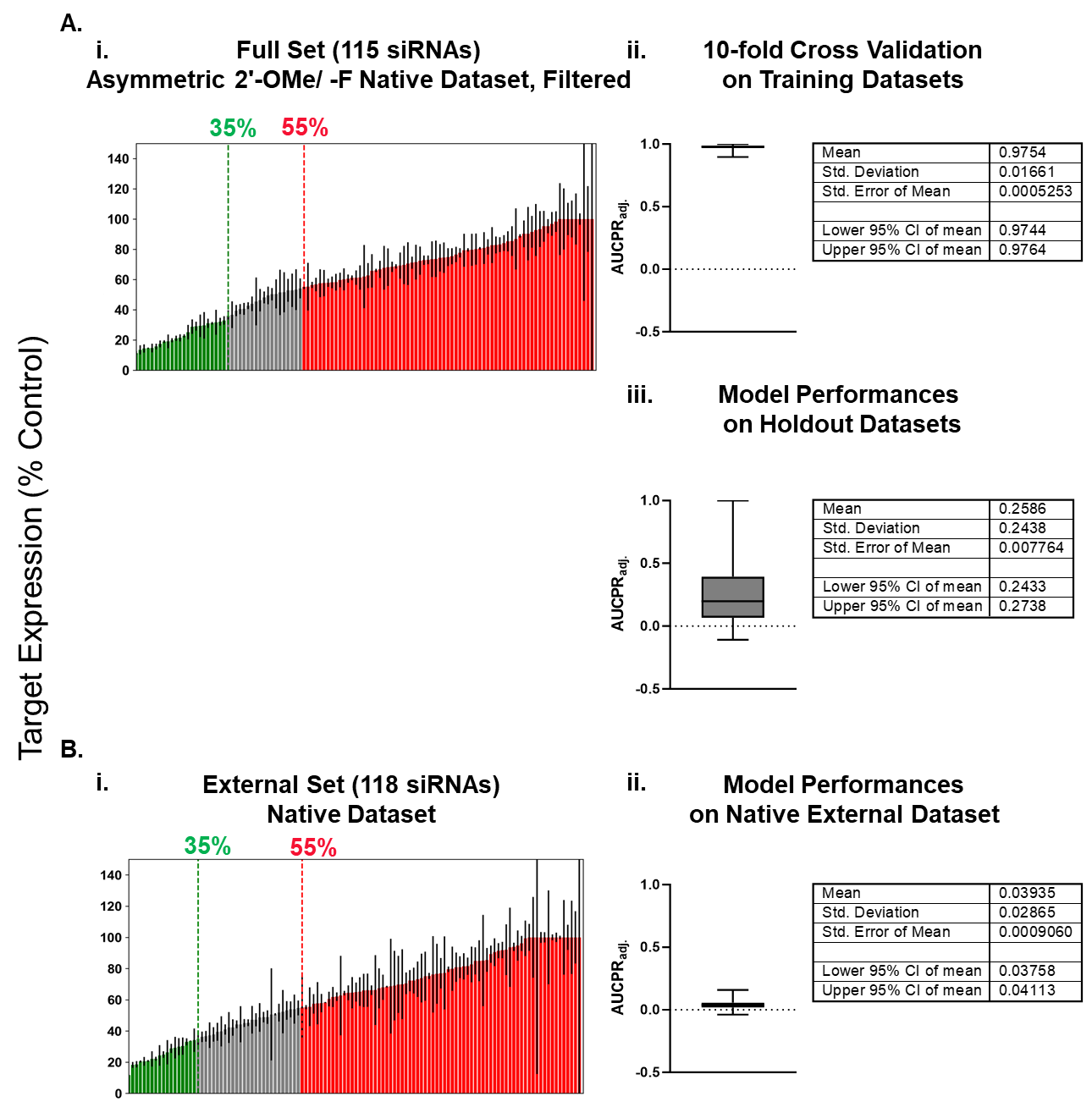
****Supplemental Figure 9. Machine Learning models trained on native assay data filtered for siRNAs targeting prominently expressed target regions poorly predict fully chemically modified siRNA efficacy for other targets in external dataset**

(A and B, left panels) siRNA (Asymmetric 2′-OMe/-F scaffold) target silencing results (n=3, mean ± SD) for (A) the full dataset in a native context used to create the models and for (B) the external dataset excluded from model building derived from native assay. Cells treated for 72 hours. Target mRNA expression levels measured using the QuantiGene 2.0 RNA Assay and calculated as percentage of untreated control. Dotted lines mark thresholds used for effective and ineffective siRNAs. (A and B, right panels) AUCPR_adj._ values plotted and statistics shown for each of the models generated from the training datasets (85% of the full dataset) for (Aii) 10-fold cross validations on the training datasets, and (Aiii) final model performances on the holdout datasets (15% of the full dataset) and (Bii) native assay derived external dataset.

**
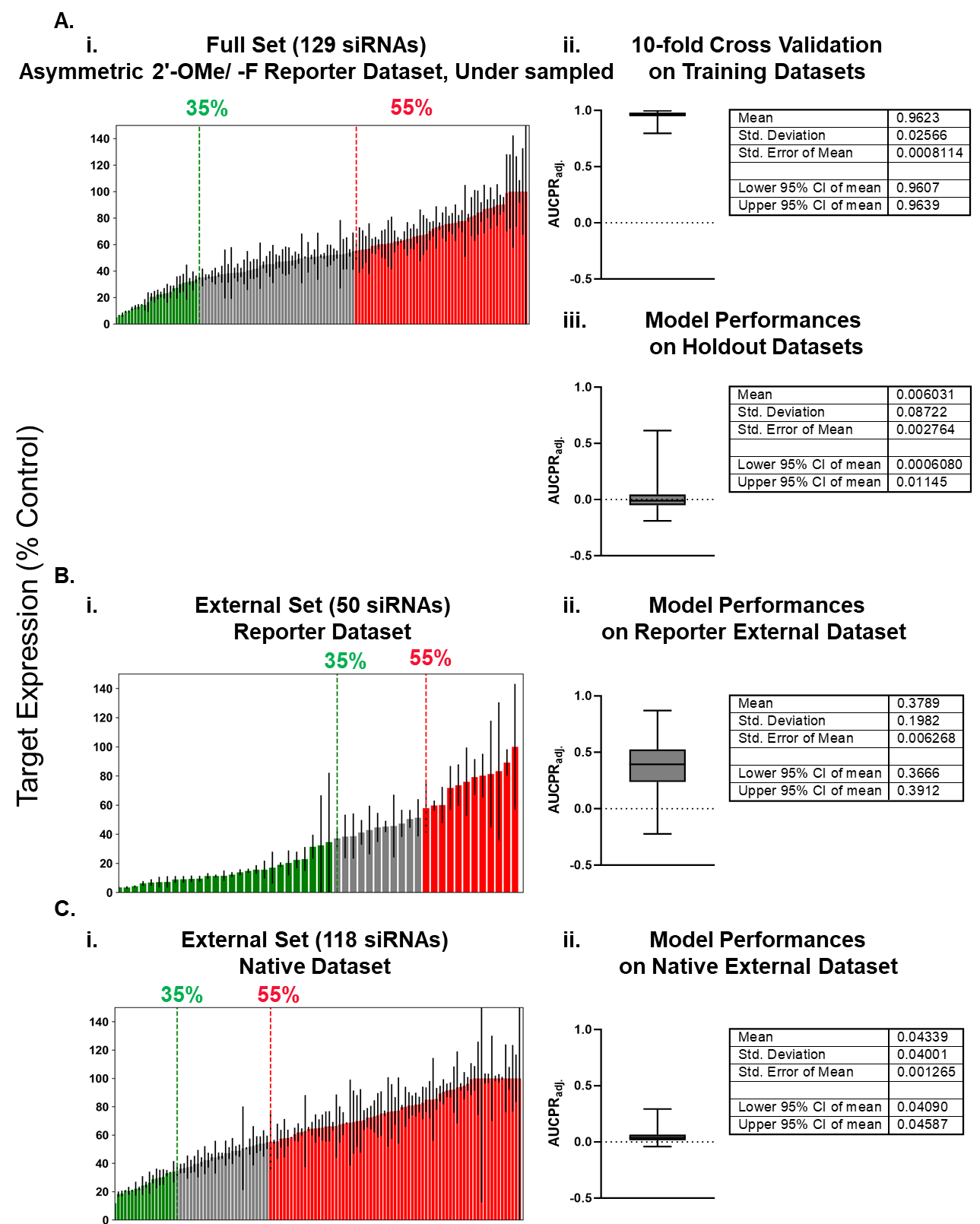
**

**Supplemental Figure 10. Machine Learning models trained on reporter assay data under sampled to match the proportion of true hits in the native, filtered dataset predict RISC-competence for fully chemically modified siRNAs**

(A-C, left panels) siRNA (Asymmetric 2′-OMe/-F scaffold) target silencing results (n=3, mean ± SD) for (A) the full dataset in a reporter context used to create the models and for (B-C) external datasets used to evaluate the models from (B) reporter or (C) native assays. Cells treated for 72 hours. Target expression levels measured using the QuantiGene 2.0 RNA Assay (native) or Dual-Glo® Luciferase Assay System (reporter) and calculated as percentage of untreated control. Dotted lines mark thresholds used for effective and ineffective siRNAs. (A-C, right panels) AUCPR_adj._ values plotted and statistics shown for each of the models generated from the training datasets (85% of the full dataset) for (Aii) 10-fold cross validations on the training datasets, and (Aiii) final model performances on the holdout datasets (15% of the full dataset), (Bii) reporter assay derived external dataset, and (Cii) native assay derived external dataset.
